# Impact of network structure on collective learning: An experimental study in a data science competition

**DOI:** 10.1371/journal.pone.0237978

**Published:** 2020-09-04

**Authors:** Devon Brackbill, Damon Centola

**Affiliations:** 1 Annenberg School for Communication, University of Pennsylvania, Philadelphia, Pennsylvania, United States of America; 2 School of Engineering, University of Pennsylvania, Philadelphia, Pennsylvania, United States of America; Unviersity of Burgundy, FRANCE

## Abstract

Do efficient communication networks accelerate solution discovery? The most prominent theory of organizational design for collective learning maintains that informationally efficient collaboration networks increase a group’s ability to find innovative solutions to complex problems. We test this idea against a competing theory that argues that communication networks that are less efficient for information transfer will increase the discovery of novel solutions to complex problems. We conducted a series of experimentally designed Data Science Competitions, in which we manipulated the efficiency of the communication networks among distributed groups of data scientists attempting to find better solutions for complex statistical modeling problems. We present findings from 16 independent competitions, where individuals conduct greedy search and only adopt better solutions. We show that groups with inefficient communication networks consistently discovered better solutions. In every experimental trial, groups with inefficient networks outperformed groups with efficient networks, as measured by both the group’s average solution quality and the best solution found by a group member.

## Introduction

Organizational communication networks are essential for solution discovery among collaborative groups. Engineers [1,2], medical researchers [3], and scientists [4] all rely on networks of colleagues and collaborators to discover innovative approaches to complex problems. Communication networks can be particularly important for organizations that face challenging search environments, in which group members must invest time and resources to discover and evaluate unproven ideas. To mitigate the costs and potential risks associated with these search efforts, organizations can invest in creating a dense network of ties among collaborators and colleagues to facilitate the discovery process [5,6]. We focus on collective learning environments where individuals face strong incentives to show continual solution improvement at each step. These include business contexts with social pressure for continual improvement, fields with well-established standards and best practices that discourage exploration, and cultures with norms against causing harm or where litigious practices discourage risky, new solutions.

One of the most influential theories of organizational communication has argued that the more efficient these communication networks are for information diffusion—i.e., the lower the “degrees of separation” between actors [7]–the more effective research groups will be at collaborating to discover innovative solutions to complex problems [8,9]. Here, “efficiency” refers to the average number of steps between members of the group, which is measured by the network’s characteristic path length. In support of this thesis, researchers have observed that not only does a highly efficient communication network increase the rate at which information about new discoveries can spread between group members [10–12], but it also facilitates coordination between colleagues [13,14] and reduces the search costs to find solutions discovered by others.

Despite the intuitive appeal of this theory, a contending theory argues that greater efficiency in communication and collaboration networks can unexpectedly reduce the rate of solution discovery. This theory suggests that while increasing the efficiency of an organizational network will improve the spread of existing solutions, it can also unintentionally limit the ability of group members to discover new solutions [15–18]. The basic idea behind this hypothesis is that the faster solutions of moderate quality diffuse through an organization, the more likely groups will abandon novel and unproven ideas, and settle for an existing solution rather than working to discover groundbreaking innovations [17]. Findings from the groupthink literature offer support for this idea [19,20]. This theory offers a striking prediction: Organizations with less efficient communication networks will yield greater rates of solution discovery for complex problems [17].

This paper examines the effect of network efficiency—measured by the average number of network steps between the members of the group—on the quality of the solutions that the group discovers. The theoretical and practical importance of understanding how communication networks impact an organization’s capacity to discover novel solutions to challenging problems has been widely appreciated [4,21,22]. However, the empirical evidence offers conflicting support. Previous experimental studies of how communication networks affect organizational innovation have produced findings that support both the theory of efficient networks [23], as well as the theory of inefficient networks [18,24,25]. The lack of clear evidence has raised doubts as to whether there is any direct causal connection between the efficiency of communication networks and the rate of solution discovery [26]. This skepticism is due in part to the fact that observational studies of problem solving on scientific and industrial teams have been unable to identify the direct causal effects of network efficiency on the process of solution discovery [8]. Additionally, past experimental studies of collective problem solving provided communication signals that do not clearly distinguish popularity from solution quality, and they were unable to study groups of researchers tackling complex problems within competitive real-world environments [18,23,24]. Our study implements the key elements of the theoretical model about the effect of network efficiency on solution quality. These include: 1) individuals search for new solutions in a greedy, incremental process; 2) individuals are incentivized to always improve; and 3) communication between team members consists only of information about solution quality with an ability to adopt a neighbor’s solution [17]. Relaxing elements of this model have led to conflicting results in the literature.

## Materials and methods

We addressed the difficulties of identifying the effects of network efficiency on group performance by studying the process of solution discovery among distributed groups of data scientists and statisticians, who were recruited from data science working groups and university statistics departments around the world, and who participated in a series of Data Science Competitions. The study was approved by the Institutional Review Board at the University of Pennsylvania (Protocol #821916), and online consent was obtained from participants. Our experimental study used an *in vivo* design based on previous data science competitions, such as the Netflix Prize (http://www.netflixprize.com) and Kaggle (http://www.kaggle.com), which connect global teams of data scientists to accelerate breakthroughs in machine learning, artificial intelligence, and statistical and computational analysis. Such competitions offer an ideal situation to test theories of communication efficiency on collective learning because the groups are fully distributed and the researcher can have complete control over the efficiency of group collaboration. Participants in the competitions were recruited from university statistics and social science programs and from online forums devoted to statistics and data science. They were skilled in statistics and reported taking an average of 3 upper-level statistics courses. Their assigned task in the competitions was to build predictive statistical models for complex data sets (see S1 File). They were rewarded financially based on the accuracy of their models’ predictions, up to a maximum of $10.

**Fig 1:**
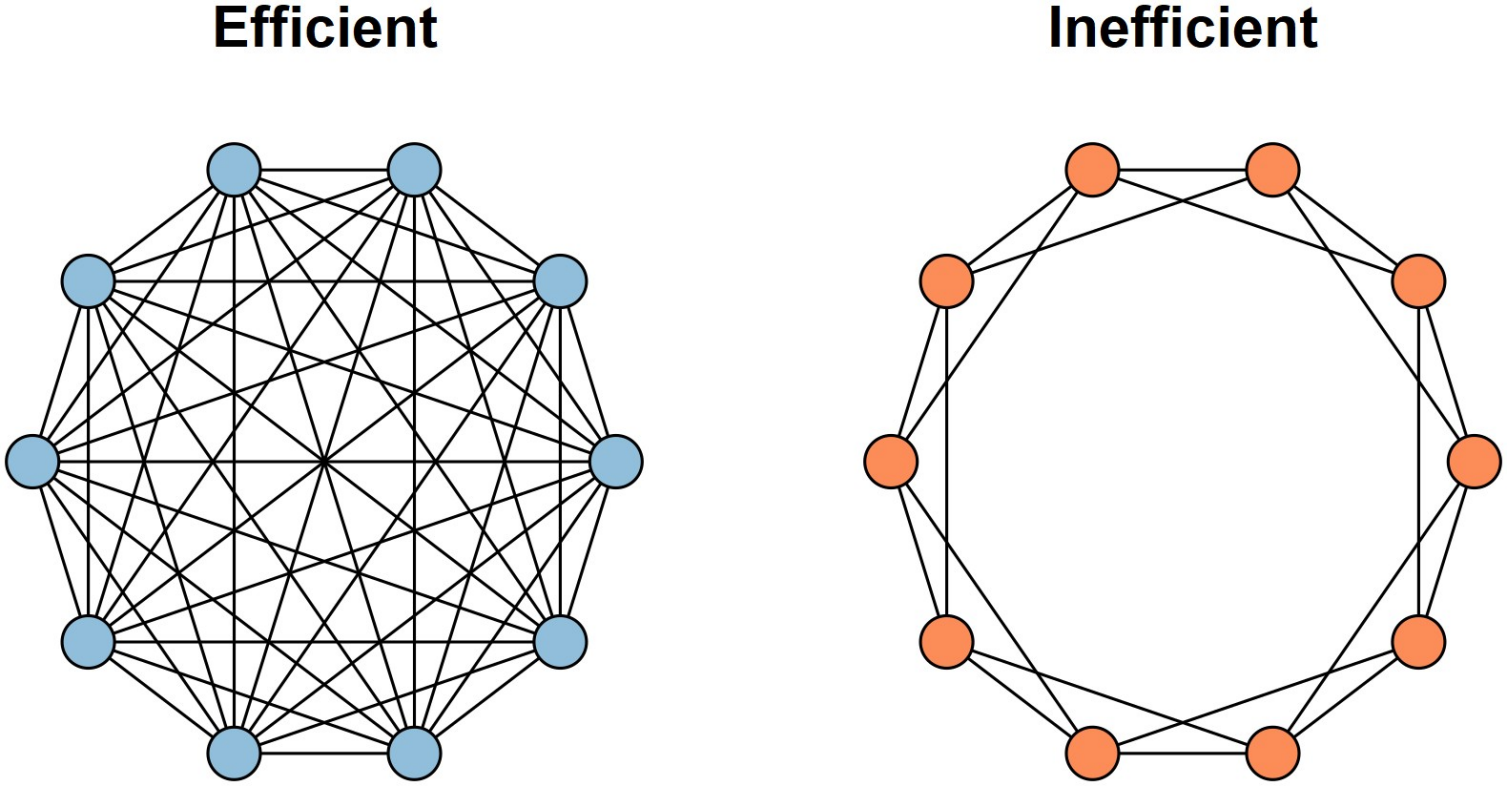
Network structures used in the two experimental conditions. The efficient network was a fully connected network (left), and the inefficient network was a one-dimensional lattice where each node was connected to its immediate four closest neighbors (right).

In each experimental trial, participants were randomly assigned to one of two groups composed of anonymous participants. The structure of the communication networks between group members varied according to experimental condition, and we focused on manipulating characteristic path length among networked groups that formed a connected component. To test the theory of network efficiency, we chose two networks that differed along the continuum of characteristic path length (see S1 File for details on the network measures). As shown in Fig 1, participants were randomized to either an efficient communication network—a fully connected network in which each participant was connected to all other members—or an inefficient network, in which participants could only see the solutions among their four immediately adjacent “neighbors” within a one-dimensional lattice topology (S1 File). Among networks with a minimum degree of 4, these networks are at opposite ends of the continuum of characteristic path length. We selected these networks because the theoretical model predicts a monotonic progression in performance as a function of path length, so if we can understand these two networks, then we can place boundaries around the impact of network structure on solution quality.

To maintain interface parity between conditions, we applied a ranking window (displayed as a “Leader Board”) to the efficient network so that participants only saw the top four solutions among all of their connections. As a result, individuals in both conditions had the same amount of information to process, but the effective path length differed between the two conditions.

We conducted 8 experimental trials, comprising 16 competitions in total. Each trial consisted of two simultaneous competitions: one group with an inefficient communication network, and one with an efficient network. 7 trials (14 competitions) were conducted with groups of size *N* = 10 (140 participants in total), and the final trial (2 competitions) had groups of *N* = 20 (40 participants in total). Overall, 180 data scientists participated in this study.

In each trial, groups of participants were given a challenging statistical modeling problem that required searching a high-dimensional space in order to find the best predictors to include in a statistical model. For instance, in one trial participants had to predict the weekly sales volume for a Fortune 500 company, and were given 14 statistical parameters to choose between, offering 16,384 potential solutions. Subjects could explore the data set using a custom research platform that was accessed via a Web browser.

Each competition lasted for 15 rounds. In each round, participants had to choose whether to test out a new solution (i.e., explore the space of solutions) by using the interactive analysis tool to submit a new predictive model, or to adopt an existing solution already discovered by another member (i.e., exploit an existing solution). To explore the solution space, participants incrementally revised their existing solution by adding or removing a variable in their model. They were able to use graphical and statistical analysis tools provided within the interface to estimate the improvements created by these changes (for screenshots of the interface see S2–S7 Figs).

Conversely, when participants decided to exploit another’s solution, their previous solution was replaced with the copied one. Thus, while exploration was incremental—i.e., participants searched locally through the complex space of potential solutions—exploitation was global—i.e., allowing participants to adopt any solution, and therefore “leap” to an entirely new part of the solution space without having to traverse the intervening steps in the complex landscape. This design accurately represents search processes used by individuals and organizations in competitive situations where exploration requires a substantial investment. In these situations, actors explore incrementally, rather than randomly exploring new ideas [6,27–29] (see S1 File). To ensure the robustness of this design, we tested this assumption using additional simulations (S1 File), which show that adding noise to actors’ decisions, thereby allowing individuals to move randomly to lower quality solutions, does not qualitatively affect the network dynamics that we report here [17].

Because our study focused on situations where individuals have strong incentives to always improve, we built several features into the interface to incentivize improvements. First, strong social signals in the form of a “Leader Board” displayed the scores of other participants in comparison to each user’s score. This informed participants that better scores were possible. Second, when participants explored a solution and the outcome was worse than their current solution, a pop-up box informed them of the new worse score, and the system returned them to their original (better) solution. Collectively, these features guided participants toward better solutions throughout the trials.

In each trial, the complexity of the problems that participants solved was defined by the ruggedness of the solution landscape [17,30]. All problems were “complex” in the sense that the statistical variables that participants could explore shared a pattern of correlations and interactions that created a high degree of interdependency among the components of each solution [30]. Consequently, any decision to include or remove one variable affected the decisions that were made about every other variable (S1 File). Consistent with most complex problems in scientific discovery and technological innovation [31], we used discrete combinatorial optimization problems that constituted “rugged fitness landscapes” [30]–i.e., there were many locally optimal solutions, which made it difficult to find the globally optimal solution (S1 File).

Each of the eight experimental trials used a unique problem. Within each trial, both groups were given the same problem. The set of starting solutions for the participants—i.e., the randomly assigned distribution of group members’ initial positions in the problem space for each group—was identical across the members of both groups, ensuring that both groups had identical initial conditions. The features of the social network, such as the average path length and the size of the population, were unobservable to participants. More generally, in every trial, all aspects of the participants’ experience were identical across experimental conditions. The only difference across conditions was the structure of the collaboration networks, which was not visible to the subjects. Eight replications of this experimental design produced eight independent pairs of competitions. Our analysis uses paired statistical tests to evaluate the effects of network structure on the quality of scientific discovery across all eight trials. This is a standard way of evaluating causal effects across replicated, pairwise experimental trials to increase the efficiency of the statistical analysis. All statistical tests were conducted at the group-level. (Further details of the statistical tests are available in the S1 File).

## Results

The results show that network structure had a significant effect on the quality of the solutions that were discovered. In every trial, groups with inefficient communication networks significantly outperformed those with efficient networks.

We used two complementary metrics to evaluate collective performance. First, we measured the best overall solution discovered within each network, which reports the highest level of innovation that each group achieved in searching the problem space. There are many “winner-take-all” situations in which groups are rewarded only for their best solution, such as an engineering team where only one solution can be implemented. Second, we measured the mean quality of the solutions among all the members of each group, which captures the diffusion of good solutions by measuring the average performance of all members of each group. There are many situations were groups are rewarded when all members perform better, such as in sales organizations where every member’s performance aggregates up to the group’s performance.

**Fig 2:**
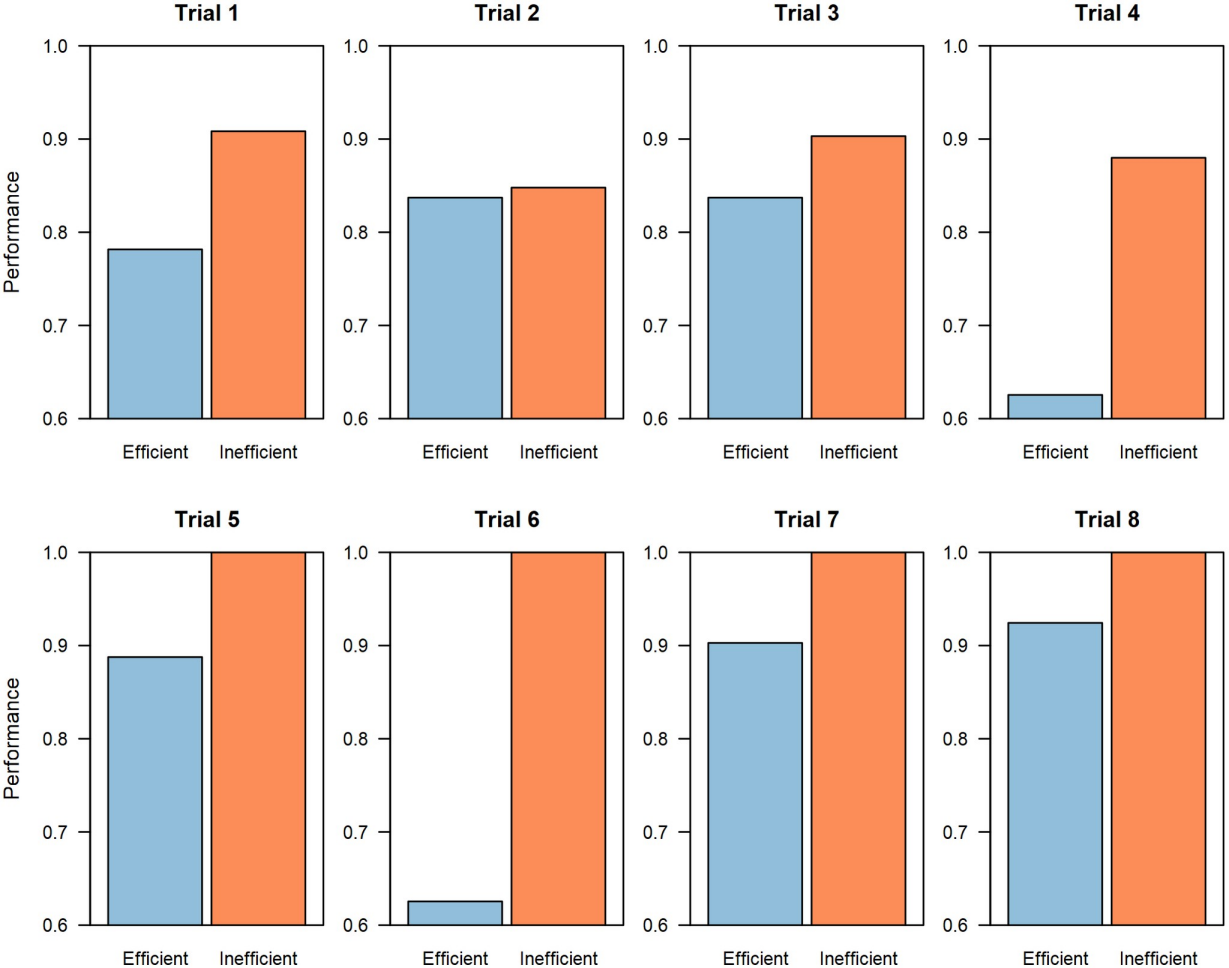
Best solution discovered in 8 experimental trials. In each of the eight experimental trials, groups with inefficient networks (orange) found better solutions than groups with efficient networks (blue). Within each trial (1–8), both groups were given the same data set and prediction problem, and began with identical initial solution distributions across the population. Across each trial, different data sets and prediction problems were used. Figures for each trial are scaled based on the best possible solution to the problem (Performance = 1) compared to both groups’ average starting performance within a trial (Performance = 0). In trials 5, 6, 7, and 8, the groups in inefficient networks found the best possible solution, which was never found in any group with an efficient network.

Fig 2 reports the best solution discovered in every trial. This value is scaled based on the best possible solution to the problem (Performance = 1) compared to both group’s starting performance within a trial (Performance = 0). The results show that by the end of the competition, inefficient collaboration networks significantly and consistently improved the best overall solutions that groups discovered in all eight trials (*p* = 0.008, Wilcoxon signed-rank test, two-sided, n_1_ = n_2_ = 8). On average, across all trials, the best solution in groups with inefficient networks was 20% better than the best solution in groups with efficient networks. More strikingly, while none of the groups with efficient networks ever discovered the optimal solution, in 50% of the trials (i.e., Trials 5, 6, 7, and 8), groups with inefficient networks found the global optimum.

To examine the effects of collaboration networks on the average quality of individuals’ solutions, in each trial we measured the average (mean) performance for the members of both groups over the course of each round. We found significant differences in the group dynamics across experimental conditions. Immediately after the initial round (i.e., Round 1) of each trial, the average solution among groups with efficient networks was 69% better than among groups with inefficient networks (*p* = 0.02, Wilcoxon signed-rank, two-sided, n_1_ = n_2_ = 8). This is because efficient networks quickly converged on the best available solution, which rapidly improved the performance of everyone in the group. However, this early advantage of efficient networks did not last long. In inefficient networks, subsequent discovery of better solutions, and diffusion of those solutions, led to rapid improvements in the average performance of all group members.

**Fig 3:**
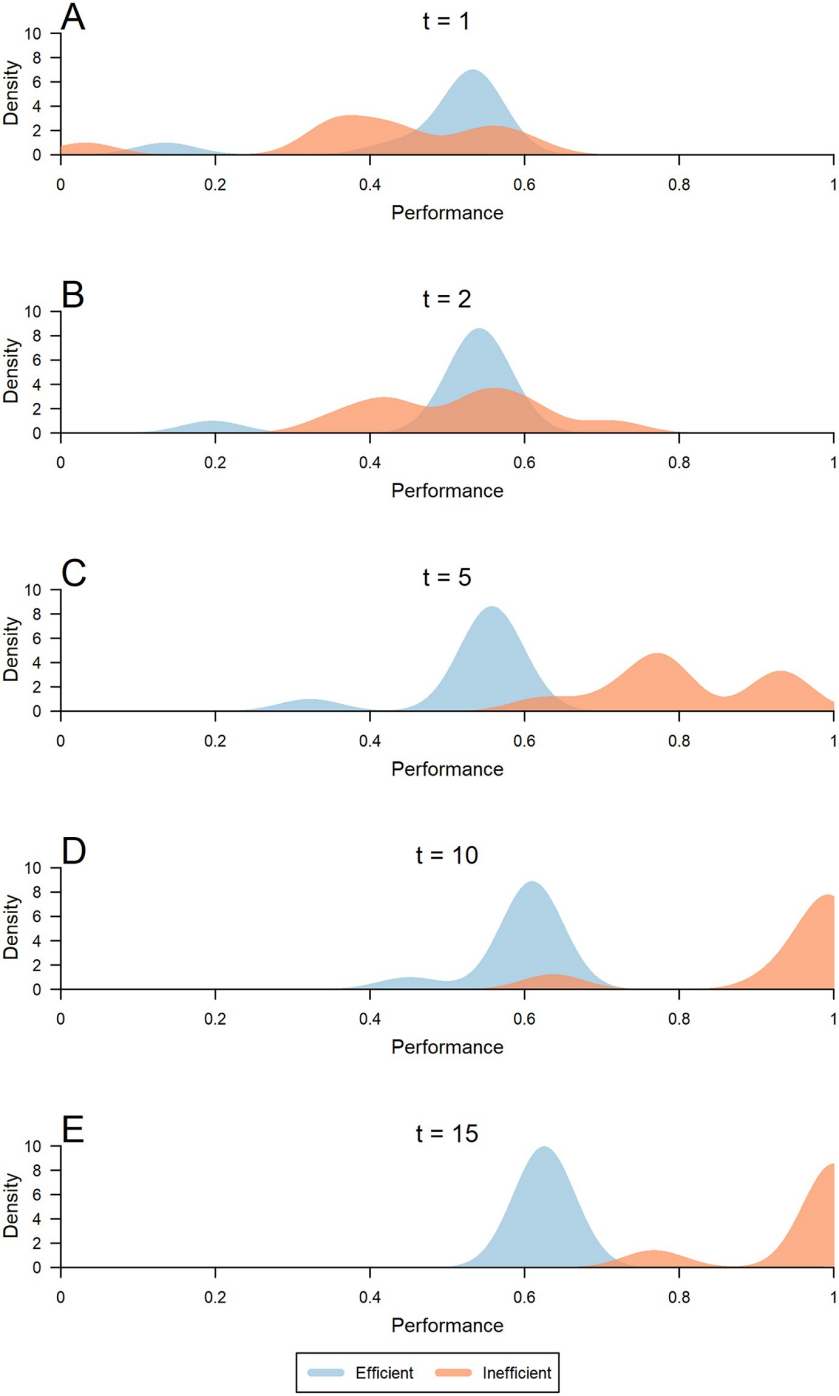
Temporal sequence of solution discovery for all members of both groups in a single trial. Solution quality in rounds *t* = 1, 2, 5, 10, and 15 (**A** to **E**) in a single trial (i.e., Trial 6). In the initial rounds, (**A**) *t* = 1, and (**B**) *t* = 2, individuals in the efficient network (blue) converged on a small set of solutions, whereas individuals in the inefficient network (orange) explored a greater diversity of solutions. (**C**) *t* = 5, the individuals in the efficient network showed little improvement, whereas individuals in the inefficient network had greater solution diversity and better solution quality. (**E**) *t* = 15, nearly every member of the inefficient network had converged on the best solution, whereas those in the efficient network showed little improvement from their initial solutions. Performance is scaled based on the best possible solution (Performance = 1) compared to both group’s starting performance (Performance = 0).

To illustrate these dynamics, Fig 3 shows the complete temporal sequence of solution discovery within a single trial (i.e., Trial 6) for both experimental conditions. In Rounds 1 and 2 (Panels A and B of Fig 3), individuals in the efficient network converged rapidly on a few solutions that were of moderate quality. In contrast, individuals embedded in the inefficient network had more diverse solutions in the initial rounds, with a lower average quality. Consequently, most members in the efficient network outperformed members of the inefficient network during the initial rounds. However, because inefficient networks were not as fast to converge, they were able to sustain a greater diversity of solutions early on. This permitted individuals to explore new parts of the problem space, including outlier solutions that outperformed any of the early innovations discovered in the efficient network. As a result of this increased diversity in the exploration space, groups with inefficient networks quickly found better solutions. Fig 3 shows that by Round 5 (Panel C), most individuals in the inefficient network had already found better solutions than the best solution discovered in the efficient network.

By Round 10 (Panel D), members of the efficient network showed little improvement from their early solutions, while a majority of participants in the inefficient network were approaching the globally optimal solution. By the end of the trial (Panel E), every member of the inefficient network adopted a solution that was better than the best solution found in the efficient network. Across all trials the same general dynamics were observed. (All eight times series are displayed in S8 Fig) In every trial, by the final round not only did the inefficient network discover a better overall solution, but the average solution among all individuals in inefficient networks was significantly better than the average solution among all subjects in efficient networks (*p* = 0.008, Wilcoxon signed-rank, two-sided, n_1_ = n_2_ = 8).

An intuitive explanation for these findings is that individuals in inefficient networks spent more time exploring, and less time exploiting, than individuals in efficient networks [23]. However, there was no difference in the fraction of exploration decisions across experimental conditions (*p* = 0.95, Wilcoxon signed-rank, two-sided, n_1_ = n_2_ = 8). Instead, differences in group performance were a result of a trade-off between solution diffusion and solution diversity. By reducing the speed at which moderately good solutions could spread early on, inefficient networks increased the overall diversity and quality of solutions that could be discovered through individuals’ explorations. Thus, while the amount of individual exploration behavior was the same in both networks, in inefficient networks, people were more widely dispersed in the solution space, so the same number of explorations produced more innovation.

**Fig 4:**
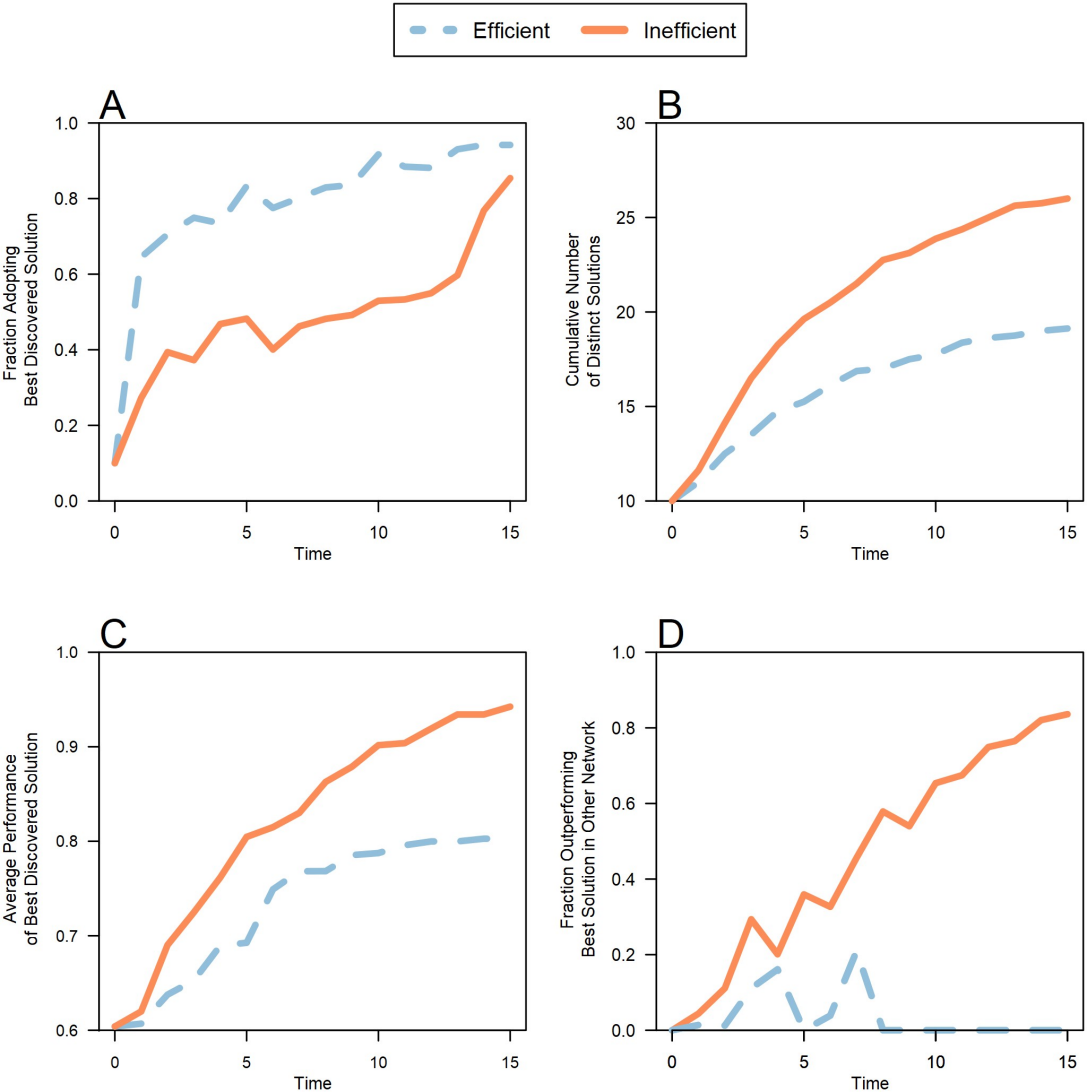
Time series showing the tradeoff between diffusion and diversity. The dynamics of solution discovery in groups with efficient (blue dashed) and inefficient (orange solid) networks shows that inefficient groups performed better in terms of solution diversity (**A** and **B**) and solution quality (**C** and **D**). All panels plot the average values for each experimental condition over all eight trials. In groups with efficient networks, good solutions rapidly spread to other group members, whereas diffusion was slower in inefficient groups (**A**). Diffusion is measured by the fraction of individuals who adopted the best available solution in the group over time. Due to this slower rate of diffusion, groups with inefficient networks discovered more distinct solutions (**B**), and the quality of the best solutions in these groups was much higher, both in terms of the value of the best solution found (**C**) and the fraction of the population that adopted a solution that was better than the best available solution from the other network (**D**).

Fig 4 (Panel A) demonstrates the speed-quality tradeoff in efficient networks. Efficient networks spread moderate solutions rapidly to the group, but they became locked in on sub-optimal solutions. The figure shows the average rate across all trials in which the best available solution diffused through each networked group. In efficient networks, the best available solution diffused rapidly. 64% of participants immediately copied this solution on Round 1. As a result, all of these participants were located in the same part of the solution space. By contrast, Panel B shows that slower diffusion of existing solutions in inefficient networks resulted in a faster rate of discovery of a greater number of solutions. Overall, groups with inefficient networks discovered a significantly larger portion of the solution space, on average finding 36% more solutions than efficient networks (*p* = 0.02, Wilcoxon signed rank test, two-sided, n_1_ = n_2_ = 8).

The consequence of this broader range of exploration (Fig 4, Panel C) is that throughout the competitions the best solution available to members of inefficient networks was, on average, better than the best solution available to members of efficient networks. As good solutions diffused, the preservation of diversity translated into a strong individual-level advantage for members of inefficient networks (Panel D). By Round 8, on average, 58% of participants in groups with inefficient networks had found solutions that were better than the best solution found in the corresponding efficient network. These dynamics of solution discovery were consistent across all eight experimental trials. By the conclusion of all trials, an average of 84% of subjects in inefficient networks had solutions that were better than the best solution found in the corresponding efficient network.

## Discussion

Our experimental results conflict with past studies of solution discovery that emphasize the importance of network efficiency both for accelerating the spread of good ideas through communities of researchers [32] and for increasing the rate of technological innovation diffusion [12]. In contrast, our study finds a tradeoff between the network structures that promote a solution’s rapid diffusion throughout a group [12,24] and the network structures that promote the discovery of innovative solutions among that group [17,18]. We found that by slowing down the information diffusion process, inefficient networks preserved the diversity of the space of exploration, thereby leading to the discovery and diffusion of better solutions. Fortunately, our results show that both “winner-take-all” situations where only the best solution is implemented and situations where the performance of each member is important in terms of mean performance provide similar support for inefficient networks over efficient ones.

Our findings suggest a cautionary conclusion concerning efforts to increase the efficiency of organizations’ communication networks [33]. Recent initiatives among organizational leaders have supported industry efforts to deploy enterprise social networking software to accelerate the speed of communications among group members. Our results indicate that while these efforts can indeed facilitate the rapid diffusion of good solutions, they may have the unintended consequence of leading to premature convergence, which can limit the exploration of complex solution spaces. As a means of managing these effects of network structure on collective learning, organizations could incentivize individuals to hold diverse and even inferior solutions. When this is impossible to institute, our results suggest another strategy: reductions in the frequency of meetings and the size of working groups can be used to moderate the effective structure of the communication networks among group members.

## Supporting information

S1 File (DOCX)

S1 Fig Schema of the experiment. (TIF)

S2 Fig Screenshot of the experimental interface when a subject explored their model. The image is similar but not identical to the experimental interface in that a university logo has been removed.
(TIF)

S3 Fig Screenshot of the experimental interface when a subject chose to copy a better solution. The image is similar but not identical to the experimental interface in that a university logo has been removed.
(TIF)

S4 Fig Screenshot of the experimental interface when a subject finished a round and adopted a better solution. The image is similar but not identical to the experimental interface in that a university logo has been removed.
(TIF)

S5 Fig Screenshot of the experimental interface when a subject finished a round and tried to adopt a worse solution. The image is similar but not identical to the experimental interface in that a university logo has been removed.
(TIF)

S6 Fig Screenshot of the experimental interface when a subject finished a round and submitted the same solution. The image is similar but not identical to the experimental interface in that a university logo has been removed.
(TIF)

S7 Fig Screenshot of the experimental interface when a subject ran out of time on a round. The image is similar but not identical to the experimental interface in that a university logo has been removed.
(TIF)

S8 Fig Solution quality a times t = 1,2,5,10, and 15 in all eight trials. (TIF)

S9 Fig Simulations showing the effects of a random noise in agents’ decisions on the performance of inefficient and efficient networks. The performance of teams relative to the best group performance (i.e., 0 probability of making a random solution choice) is plotted against the probability of making a random choice on each turn.
(TIF)

S1 Table Descriptive statistics of the data sets used in the study. (DOCX)

S1 Dataset Dataset of all user solutions and actions in the experiment. Compressed (.zip) archive containing the data set in .csv format and a README.txt file explaining the columns.
(ZIP)
