# Supplementary material for: Impact of network structure on collective learning: An experimental study in a data science competition
Source: PLoS One. 2020 Sep 4;15(9):e0237978. doi: 10.1371/journal.pone.0237978 (PMC7473554; doi:10.1371/journal.pone.0237978)
Supplement: S1 File — (DOCX) [file pone.0237978.s001.docx]

Supporting information for:

Impact of network structure on collective learning: An experimental study in a data science competition

Devon Brackbill and Damon Centola

Damon Centola

Email: dcentola@asc.upenn.edu

**This PDF file includes:**

Extended Materials & Methods

Supplementary Discussion

S1 to S9 Figures

S1 Table

References for SI reference citations

Supporting Information Text

Extended Materials and Methods

**Experiment Design.** Each trial in the experiment consisted of a comparison between two competitions— one team with an efficient network and one team with an inefficient network. In each trial, teams faced the same problem. As subjects came into a trial, they were randomly assigned to a team with one of the network conditions. The schema for this design is shown in Figure S1. Once subjects were randomized to a network condition, they were randomly assigned to one node in the network, and they maintained this position throughout the experiment. In each trial, both networks had the same size (either *N*=10 or *N*=20), but they differed in terms of their average path length. The networks also differed in terms of their degree, density, and diameter, but average path length has been identified by theoretical research as having the most impact on collective performance for the types of problems examined in this study (1). The efficient communication network was a fully connected network where each participant could receive information about the active solutions of all their teammates (characteristic path length = 1). The inefficient network was a one-dimensional ring lattice where each member could receive information from their four immediately adjacent neighbors (characteristic path length =1.67 for *N* = 10, and characteristic path length = 2.89 for *N* = 20). In total, eight independent trials were run. Across all eight trials, half of the subjects were randomly assigned to the efficient network condition and the remaining half were enrolled in the inefficient network condition. By holding all features of the design constant except network structure, we can causally identify how network structure affects the quality of solutions each team discovers.


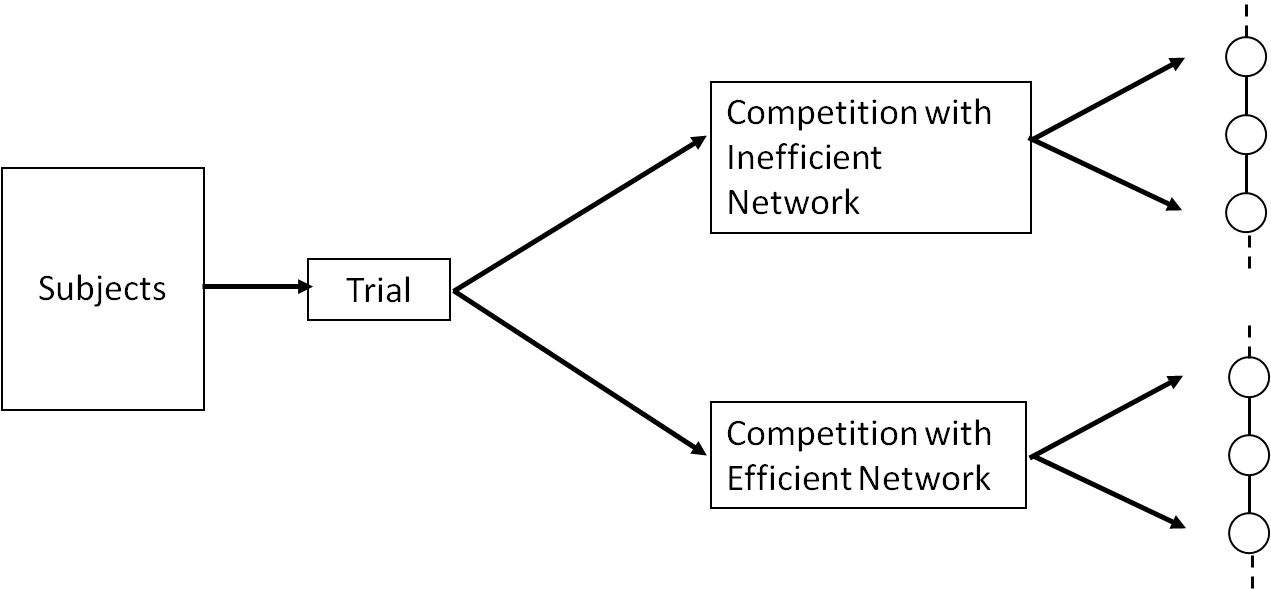


**S1 Fig. Schema of the experiment.** Subjects who arrived for a trial were randomly assigned to a competition with an inefficient or efficient network structure, and then randomly assigned to a single node within the network, where they remained throughout the study.

**Subject Recruitment.** Participants in our study were recruited via online advertisements posted on the World Wide Web to participate in a “Data Science Competition.” When subjects arrived to the study website, they registered to participate by completing a form that required them to submit their email address, and choose an avatar and a username. All participants were required to provide informed consent in order to complete the registration process.

Advertisements were placed online and direct emails were sent to several thousand interested participants. This recruitment campaign generated 1,182 unique registrations in the system. From this pool, we invited subjects back to participate in a competition on a specific date. By following a link that only became active shortly before the competition began, participants could access the online platform at the specified date and time. When participants arrived at an active competition, they viewed instructions on how the competition worked, and waited until other participants arrived. When a sufficient number of subjects arrived to conduct a single trial of the study (i.e. 20 individuals), all 20 subjects were then randomized to experimental conditions as described above, at which point the competitions would begin in both conditions. The study was run for a 127-day period, December 10, 2015 through April 14, 2016, over which time subjects were continually recruited to participate in the study. In total, 180 unique subjects participated in the study. Of the 180 individuals in the main study, 90 participated in inefficient networks, and 90 participated in efficient networks.

**Subject Pool.** Due to the difficulty of the task participants faced in the study, we recruited subjects who were specialists in statistics. We wanted individuals who worked on these complex problems, so that we could capture the behavior of teams of problem solvers. The subjects in the study were skilled in statistics and quantitative methods. In order to participate, subjects had to understand how to run a linear regression model, and how to interpret coefficients, p-values, and model performance. While subjects did receive an introductory video, this video only described how the platform interface worked, and it did not include instructions about statistics. As a result, subjects were informed that the competition would be demanding, and that they should only participate if they had a firm grasp of statistics. All online recruitment efforts were directed toward individuals with quantitative skills. To assess the statistical training of the sample, we provided a voluntary survey question that asked how many statistics courses each participant had taken. Of the individuals who responded to this question, the participants took a mean of 3 (SD=3.6) college-level statistics courses.

**Subject Experience During the Experiment.** To isolate the causal effect of network structure, the interface in each condition was identical. Individuals began a competition with a randomized initial solution. Then, individuals decided to revise their current solution by adding or removing a single variable (i.e., explore), or they decided to adopt a solution from one of their neighbors (i.e., copy). Subjects made the decision to explore or copy by clicking on a radio button on the right side of the interface that allowed them to select their own solution, or select another player’s solution. The interface when subjects selected to revise their own solution is shown in Figure S2. Subjects could add or remove one variable by clicking the button with the variable’s name. When they had made their choice for the round, they had to press the red “Submit” button on the right side of the interface. When a better option was available to copy, subjects saw the interface in Figure S3, which included a pop-up box to indicate the better model. To copy a neighbor’s model, individuals had to click on the radio button next to their model and then press the “Submit” button to end the round. The interface displayed the option to copy only when one of the individual’s neighbors had a better solution. On rounds where the individual had the best solution in their neighborhood, the interface defaulted to showing the interface to explore their own model in Figure S2.

After submitting their decision to either explore or copy for a round, individuals received their solution’s score, and they waited until other players finished the round. When individuals found a better solution, they received a pop-up tracking their improvement as shown in Figure S4. When individuals tried a new solution, but it was not better than their previous solution, they received a notification that they would be returned to their previous solution as shown in Figure S5. If individuals decided to submit the same solution, they received a notification indicating their choice as shown in Figure S6. Finally, if individuals made no choice before the timer ran out, they would remain at their previous model, and they received a notification as shown in Figure S7. These were the only possible states that participants could occupy throughout the competition.

In each competition, this sequence of events was repeated for 15 rounds in total, and each round lasted for 1 minute with an additional 5 to 10 seconds between each round. Prior to each competition, participants viewed an instruction video that introduced the interface and specified the goals of the competition. The entire experiment lasted about 20 to 25 minutes in total. We registered every click on each round—either decisions to explore or copy—so we had complete records of individual decisions. To motivate subjects, rewards were based on the quality of their final solution with a maximum payout of $10. Additionally, social motivation was triggered throughout the competition with the placement of a ranked list of performance on the top right of the interface. After the competition was finished, participants saw a final ranking showing the performance of all participants and their earnings.


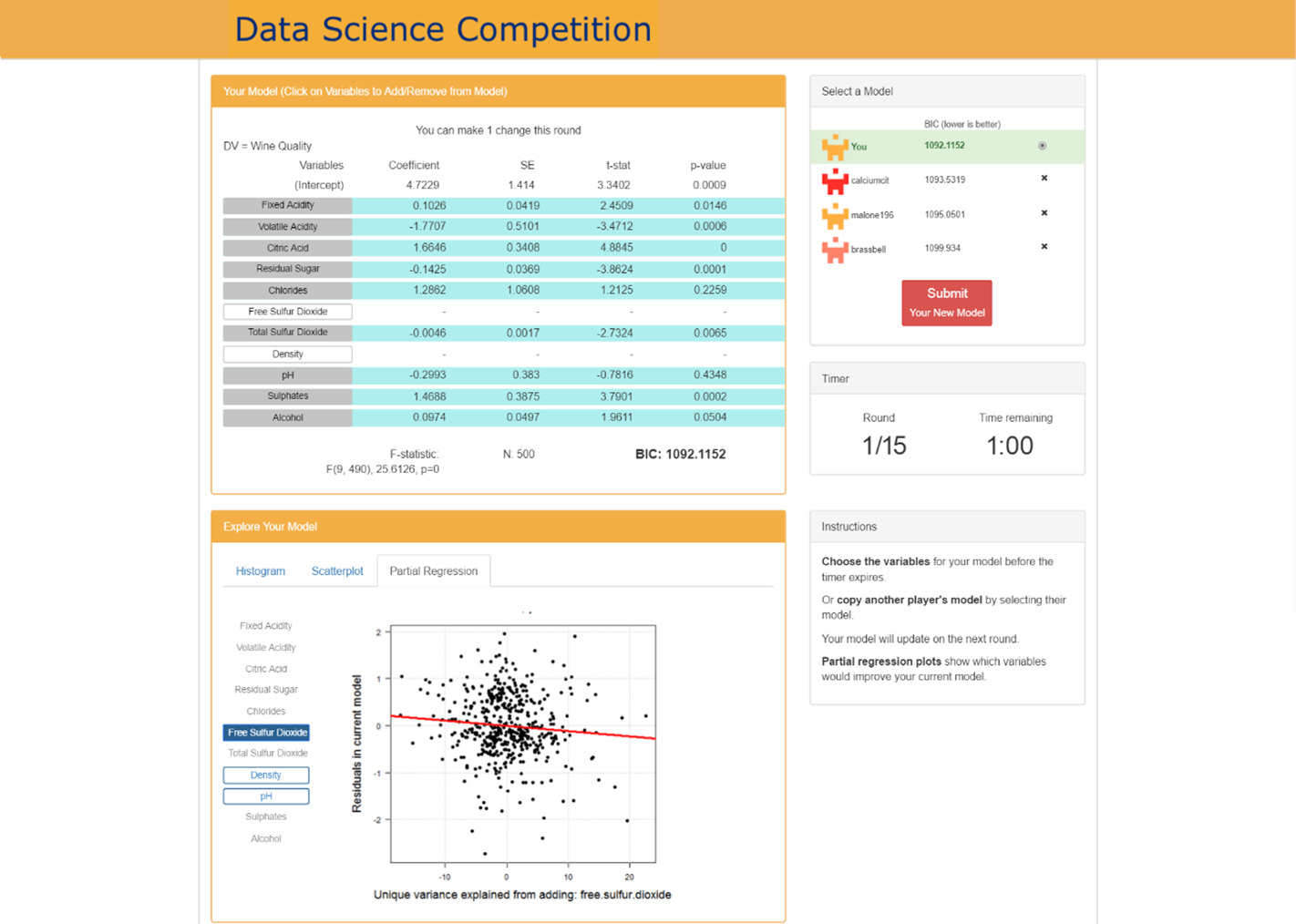


**S2 Fig. Screenshot of the experimental interface when a subject explored their model.** The image is similar but not identical to the experimental interface in that a university logo has been removed.


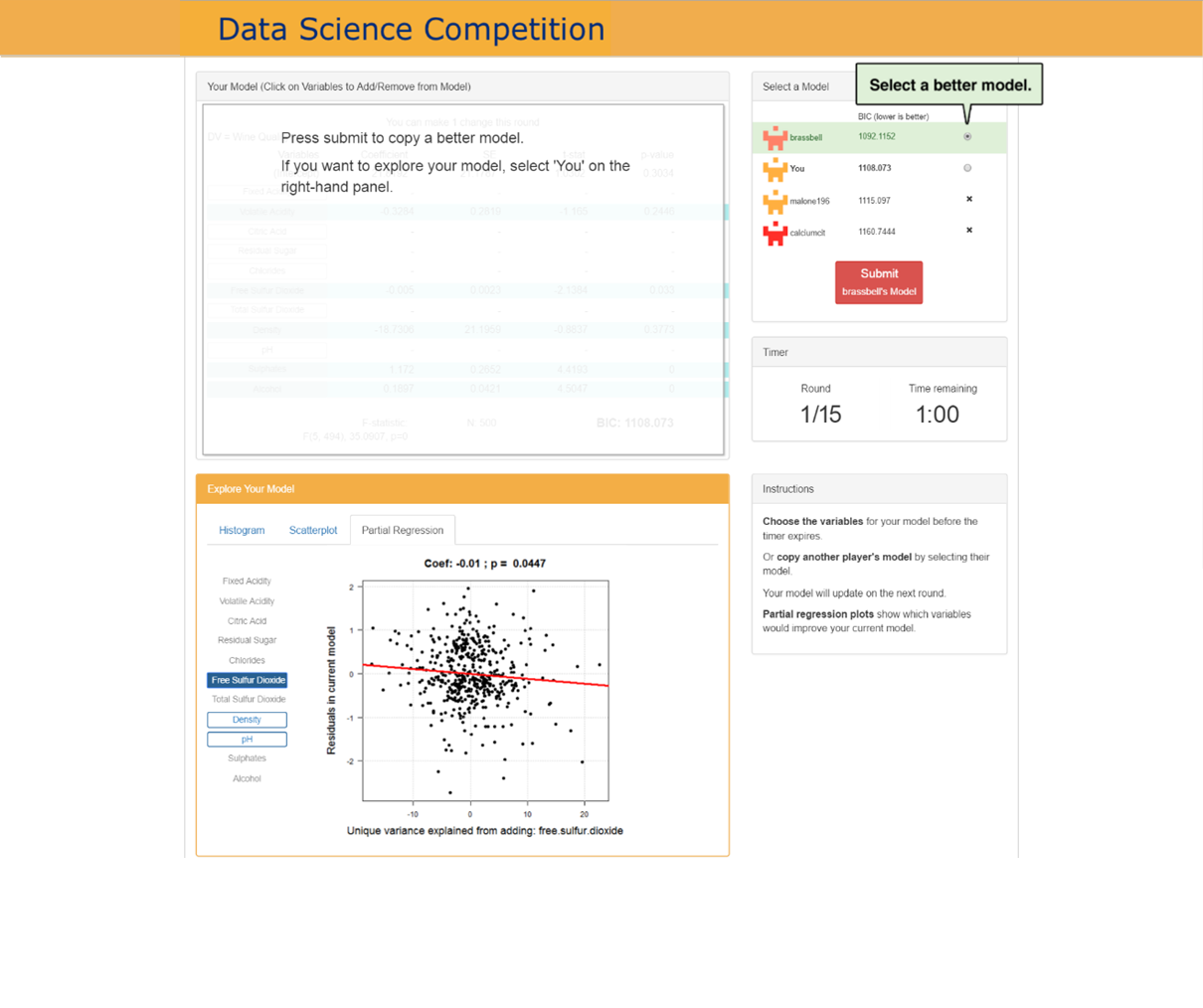


**S3 Fig. Screenshot of the experimental interface when a subject chose to copy a better solution.** The image is similar but not identical to the experimental interface in that a university logo has been removed.


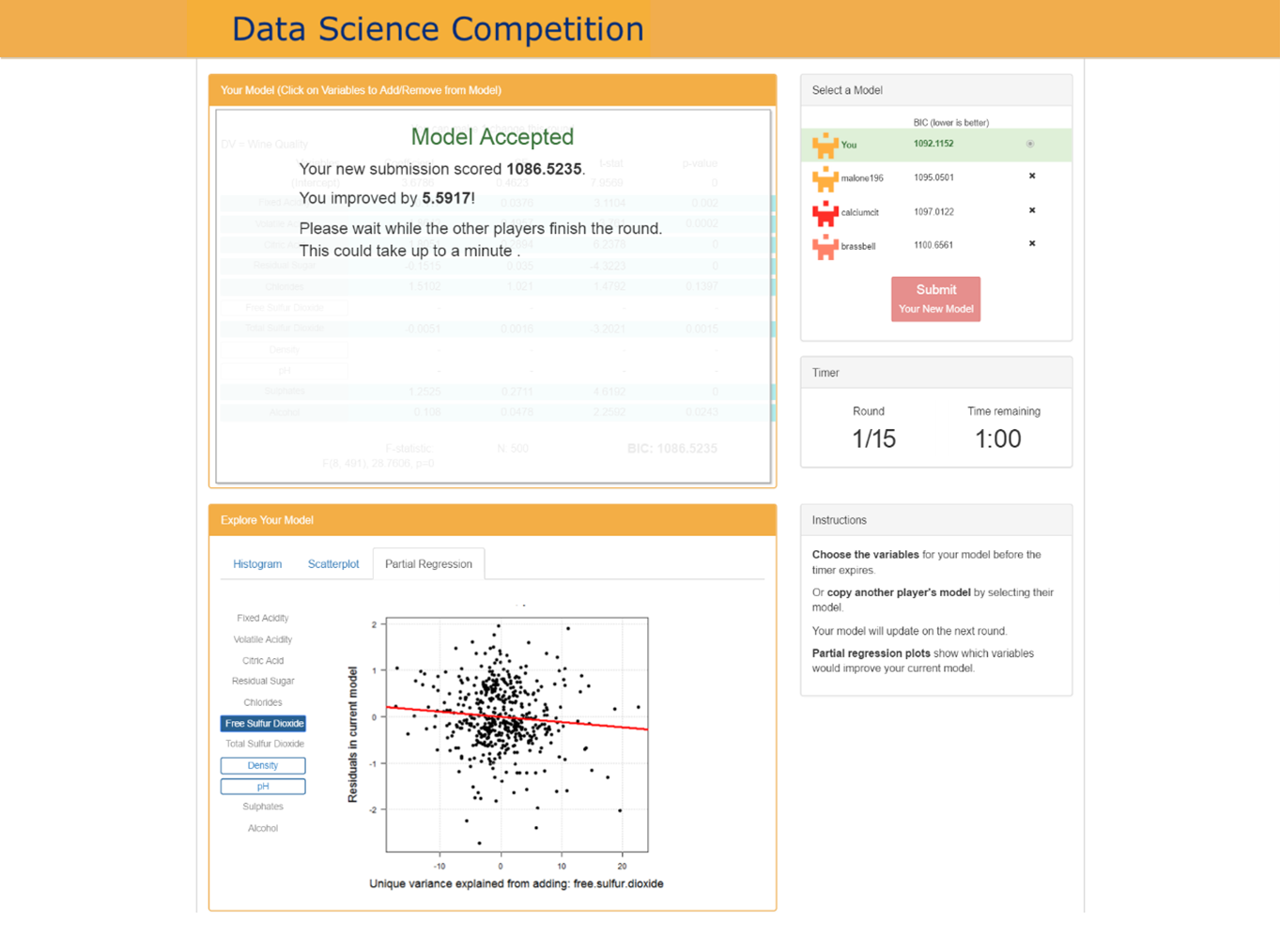


**S4 Fig. Screenshot of the experimental interface when a subject finished a round and adopted a better solution.** The image is similar but not identical to the experimental interface in that a university logo has been removed.


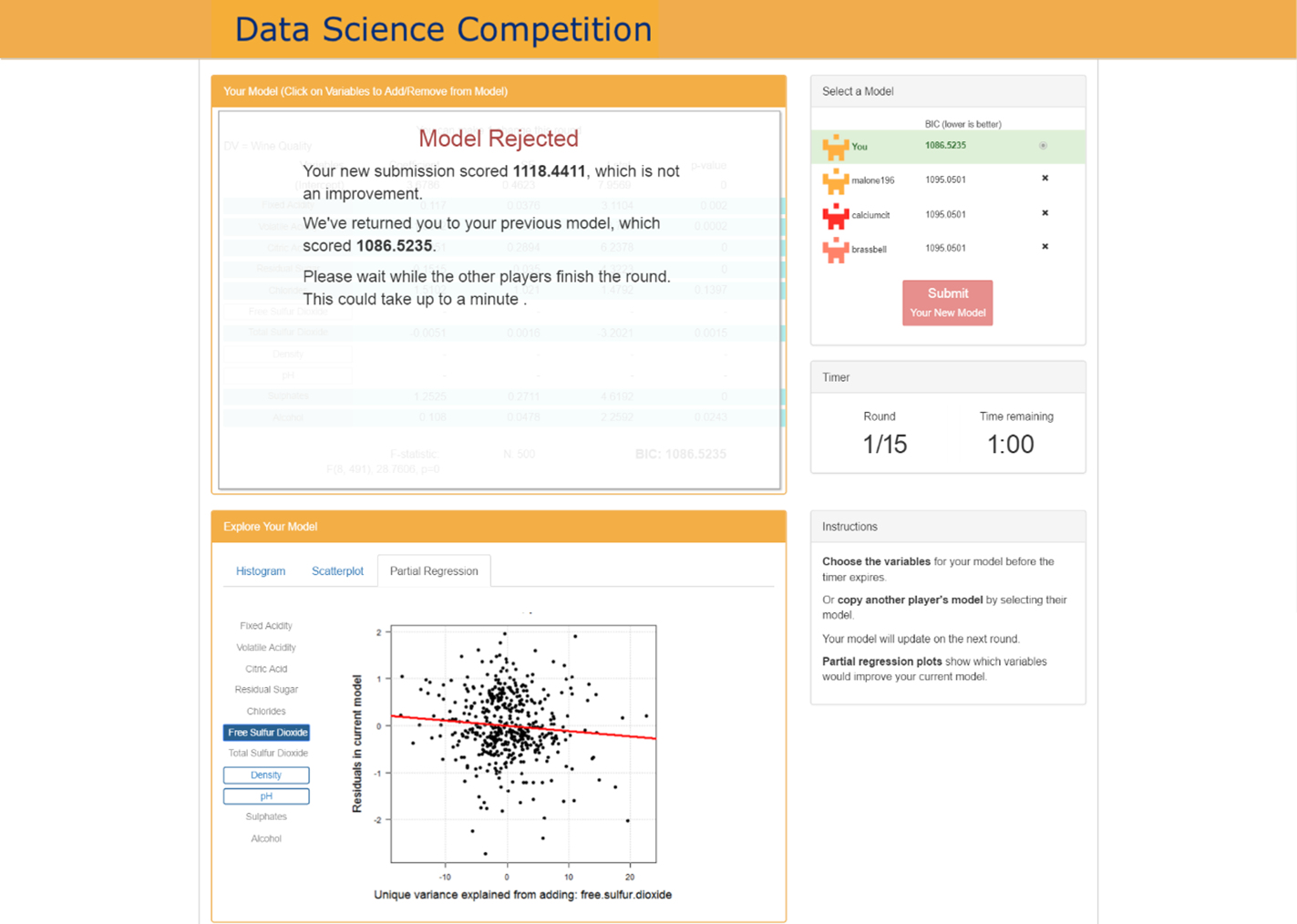


**S5 Fig. Screenshot of the experimental interface when a subject finished a round and tried to adopt a worse solution.** The image is similar but not identical to the experimental interface in that a university logo has been removed.


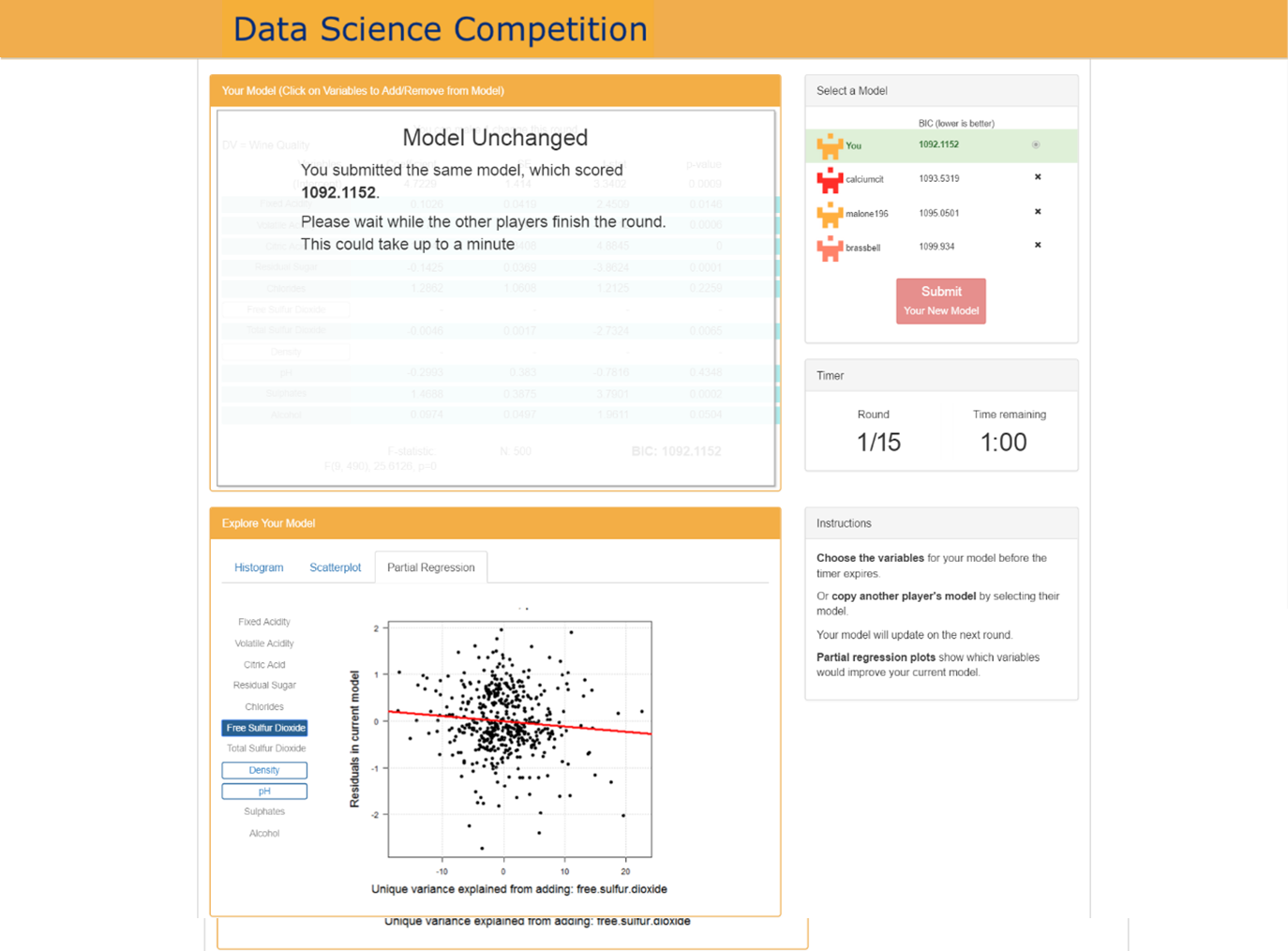


**S6 Fig. Screenshot of the experimental interface when a subject finished a round and submitted the same solution.** The image is similar but not identical to the experimental interface in that a university logo has been removed.


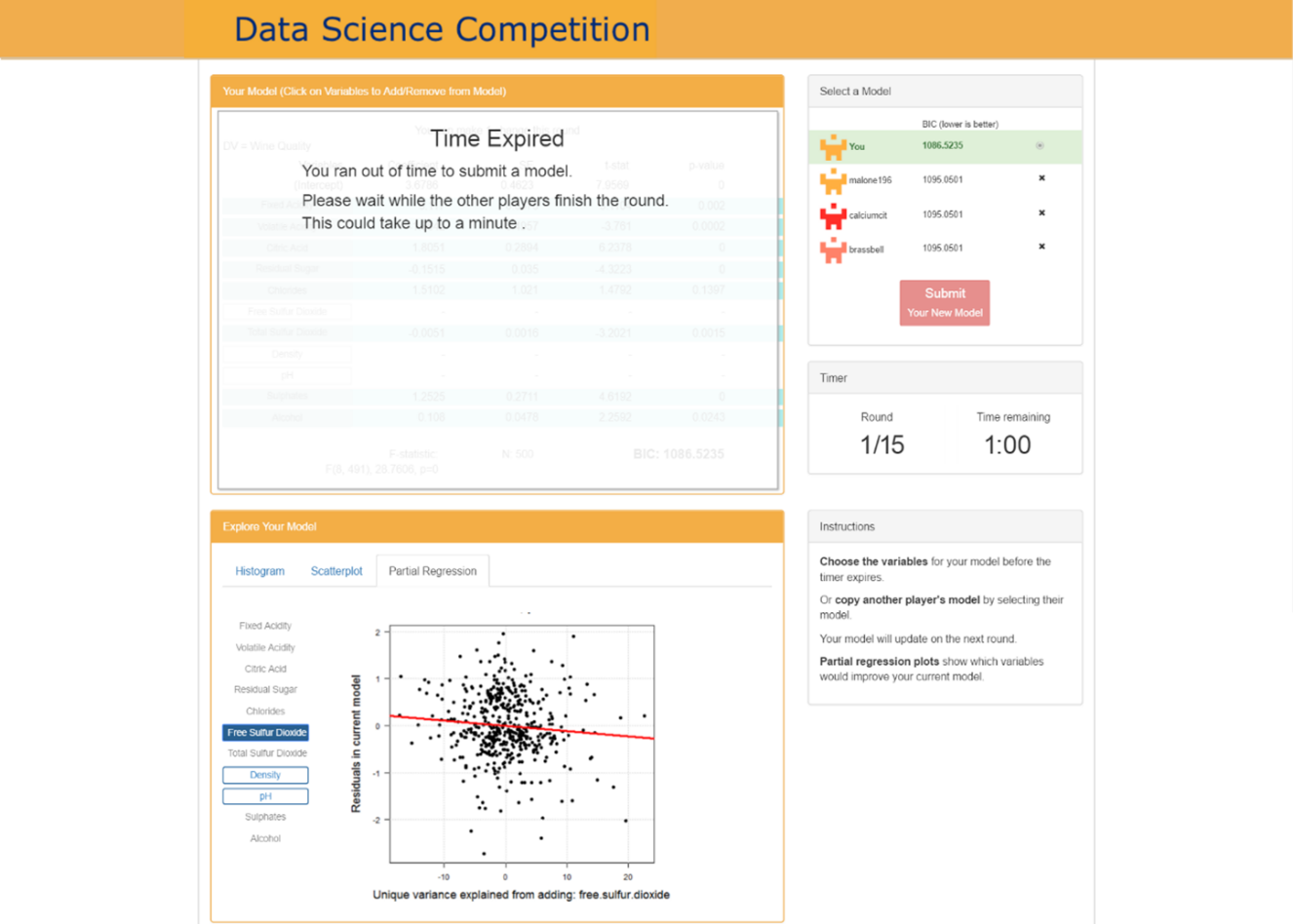


**S7 Fig. Screenshot of the experimental interface when a subject ran out of time on a round.** The image is similar but not identical to the experimental interface in that a university logo has been removed.

**Structure of the Complex Problems.** Each experimental trial involved a network of individuals who were invited to compete on a platform designed specifically to host a data science competition and to study this research question. Solutions were evaluated on each round based on the Bayesian Information Criterion (BIC) of their model, which provided continual feedback on their performance at each round. While we did not evaluate models based on their out of sample performance, as is common in data science competitions, we instead used the BIC because it is a good asymptotic measure of out-of-sample model performance and performs similarly to cross-validation predictive accuracy (2). The BIC rewards constructing sparse models that do not overfit to the current data set but that generalize to other data sets that were created by the same data generating mechanism. Individuals began with a randomly assigned model and could explore from that point.

In order to ensure that the problems contained sufficient complexity, we used a method of creating data sets that draws directly on the *NK* model, which is a model of complex problems (3). Complex problems exhibit interdependency among the components of a solution—i.e., changing one dimension affects the fitness contribution of another dimension (3). Such synergies among the components of a solution produce many local maxima in the problem space so that incremental, local search can miss the global maximum.

Using the BIC score allowed us to create complex problem landscapes where adding and removing a variable could produce surprising complex patterns. In contrast to $R^{2}$, which will always improve with the addition of more variables, performance metrics such as BIC, adjusted $R^{2}$, and out of sample performance scores do not always improve with more variables. Instead, these measures penalize adding variables that do not provide information beyond those variables already in a model. Thus, we created fitness landscapes where adding one variable would only improve the model performance if a second variable was also included. In the absence of that second variable, the addition of the first one would harm the model’s performance. In short, we created complex interactions among the predictor variables in each data set so participants faced difficult combinatorial optimization problems with many local maxima.

In a simple problem, where variables do not interact, each predictor variable contributes statistically independent information that improves the model’s quality. When a predictor variable explains no variance in the dependent variable, its contribution will be clear because the BIC will decline with this variable’s addition. In contrast, in a complex landscape such as most real world data sets, there are correlations among the variables, and each predictor variable will contain information about the dependent variable that is unique to that predictor variable as well as information already captured by other variables. As a result, some variables may only add redundant information, which will reduce the BIC score and lead to worse out-of-sample performance due to the model learning idiosyncratic information about that particular random variable that is not generalizable to unseen testing data sets (4).

To ensure that the data sets in our study had a complex structure with many interdependencies among the variables, we adopted existing data sets and increased the correlations among the variables. Each original set of continuous predictors, **Z**, was transformed into the final data set, **X**, using the following procedure:

1. standardize the matrix **Z** by subtracting each element by its column mean and dividing by its column standard deviation;
2. create a matrix, **X**, where each column in **Z** is added to the next variable in the following way, thus increasing the interdependency among the variables:

$$\boldsymbol{X}_{\boldsymbol{j}}=\frac{\boldsymbol{Z}_{j}+\boldsymbol{Z}_{j+1}}{\sqrt{2}}$$

(The final variable is simply divided by $\sqrt{2}$);

1. return **X** to the original scale of **Z** so that it looks like the original data and provides plausible values by multiplying  **X** by the column standard deviation in **Z** and adding the column mean in **Z**.

This procedure creates a data set with several important properties. First, the variables in **X** have similar summary statistics, such as means and standard deviations, as compared with the original data set, **Z**. Second, the same variables in the original data set, **Z**, will also be predictive in the transformed data set, **X**, so that domain knowledge will be very useful in the competition. We verified that the data sets felt realistic to participants in a pilot study. Thus, participants’ ideas and intuitions about real world variables will also hold true in the adjusted data sets. Finally, there will be additional correlation between the variables so that the contribution of the *j*th variable will depend upon the presence or absence of the *j+1*th variable. This pattern of dependency among variables creates a complex problem landscape that is a direct instantiation of the *NK* model (3).

We applied this procedure to create three data sets that were used throughout the eight competitions in the study. In each competition, the columns of the final data set, **X**, were randomly shuffled so that participants could not determine the pattern of correlations. To ensure that the problems were sufficiently complex, we ran an exhaustive search of all possible linear regression models, and then counted the number of local optima in the solution space. A solution was considered a local optimum when adding or removing any single variable would result in a worse solution. A simple problem should have one local optima, whereas a complex problem has several. The data problems were large and complex, with 2,048 to 16,384 possible solutions and 9 to 16 local optima, as shown in Table S1. The data sets were retrieved from the UCI Machine Learning Repository (5).

|  | |  | | | | | |  |  |
| --- | --- | --- | --- | --- | --- | --- | --- | --- | --- |
| Competition | Data Set | | | Variables (no.) | Total Solutions (no.) | Variables in Optimal Solution (no.) | Local Optima  (no.) | Variables in Local Optima (mean) | Obs.  (no.) |
| 1 | Wine | | 11 | | 2,048 | 5 | 11 | 6.7 | 500 |
| 2 | Online News Popularity | | 14 | | 16,384 | 4 | 9 | 4.6 | 500 |
| 3 | Online News Popularity | | 14 | | 16,384 | 4 | 9 | 4.6 | 500 |
| 4 | Sales Forecast | | 14 | | 16,384 | 7 | 16 | 5.9 | 1000 |
| 5 | Sales Forecast | | 14 | | 16,384 | 7 | 16 | 5.9 | 1000 |
| 6 | Sales Forecast | | 14 | | 16,384 | 7 | 16 | 5.9 | 1000 |
| 7 | Sales Forecast | | 14 | | 16,384 | 7 | 16 | 5.9 | 1000 |
| 8 | Sales Forecast | | 14 | | 16,384 | 7 | 16 | 5.9 | 1000 |

S1 Table. Descriptive statistics of the data sets used in the study.

**Network Metrics.** Average path length (or characteristic path length) is the mean geodesic or shortest path connecting two pairs of vertices (6). It is defined as the following for undirected graphs:

$$L=\frac{1}{n}\sum_{i\in N} L_{i}=\frac{1}{n}\sum_{i\in N} \frac{\sum_{j\in N,j\neq i} d_{i,j}}{n-1}$$

where *L_i_* is the average distance between node *i* and all other nodes; *d_i,j_* is the shortest path connecting nodes *i* and *j*; and *n* is the population size. It is a measure of the efficiency with which information can flow through a network. Higher path lengths indicate less efficient communication networks, and lower path lengths indicate more efficient information spread. We use the undirected, unweighted version of this metric because the networks used in our study were of this nature.

**Data Analysis.** The experimental design consisted of eight independent pairs of competitions—one efficient and one inefficient network. We used paired statistical tests to evaluate the effects of network structure on the quality of solution discovery across all eight trials, which is the standard way of evaluating causal effects across replicated, pairwise experimental trials (7). All statistical tests were conducted at the group-level.

The performance of each model was measured in terms of its Bayesian Information Criterion (BIC), which is also known as the Schwarz Criterion (8). The BIC is a measure of a model’s accuracy on a new sample of data that the model has never seen before. The BIC is calculated as the sum of two components: the likelihood function and a regularization term that penalizes the addition of more parameters. The BIC is defined as:

$$BIC=-2\bullet\ln\hat{L}+k\bullet\ln(n)$$

where $\hat{L}$is the maximum of the likelihood function of the model, *k* is the number of free parameters to be estimated, and *n* is the number of observations in the data set. In the case of the linear regression models used in the experiment, *k* is the number of regressors including the intercept in the model. Like most performance metrics, the BIC trades off the accuracy of the model against the possibility that the model has been overfit to the data as captured by the number of parameters in the model. The best model minimizes the BIC score.

To create a measure of team performance that could be compared across all trials, we rescaled the BIC metric onto the range [0,1] so that 1 indicated the best model performance. Since lower BIC values indicate a better model, we created a measure of a team’s best overall performance by inverting the BIC as follows:

$Best_{i,t}=\frac{mean_{i,t=0}(BIC)-\min_{i,t}(BIC)}{mean_{i,t=0}(BIC)-\min^{*}(BIC)}$,

where the numerator is the difference between team *i*’s average starting BIC on the initial round (*t=*0) and the best (minimum) BIC adopted by a member on the current round *t*, and the denominator is the difference between the team’s average initial BIC and the best possible solution (i.e., the global minimum). We constructed the mean team performance in a similar manner according to the following formula:

$$Average_{i,t}=\frac{mean_{i,t=0}(BIC)-mean_{i,t}(BIC)}{mean_{i,t=0}(BIC)-\min^{*}(BIC)}$$

where the numerator is the difference between team *i*’s average solution at *t* = 0 and the team’s average at time *t*, and the denominator is the difference between the team’s average initial BIC and the best possible solution. Both metrics range from 0 (the team’s initial mean solution) to 1 (the best possible solution), and indicate how much teams have improved from their initial starting mean solution toward the best possible solution.

The average initial starting solution was fixed between network conditions within a given trial because each efficient network received the same distribution of initial solutions as its corresponding inefficient network. As a result, this metric is directly comparable between conditions so long as teams started with the same distribution of initial solutions and used the same data problem. Thus, we assess the effect of the network structure using a matched pair design, comparing teams within each trial that differed only in their network structure.

To assess the performance of the best solution each team found, we plotted the best solution on the final round, *Best_i,t=15_*, for each network structure in the eight trials in Figure 1 of the main text. We conducted a statistical test of the difference between conditions using a Wilcoxon signed-rank test. This test is a non-parametric test for matched pairs comparing the probability that observations from one condition will be greater than those from another condition. In essence, it tests whether it is more likely than chance within each matched pair that one network structure will consistently have a higher performance than the other. It is very similar to the paired *t*-test, but it provides a more conservative estimate of significance because it does not assume a normal distribution of solutions. We rejected the null hypothesis that the differences between conditions was the result of chance with a probability of *P* < 0.01. All statistical tests used a two-sided test of significance.

To examine the average performance of teams, we compared *Average_i,t_* in each condition using the Wilcoxon signed-rank test. We conducted this test on the initial round when efficient networks had higher mean solutions (*t* = 1). We rejected the null hypothesis that there was no difference in the mean solution across conditions with a probability of *P* = 0.02. We also conducted this test on the final round when inefficient networks had higher mean solutions (*t* = 15). We rejected the null hypothesis that there was no difference in the mean solutions across conditions on the final round with a probability of *P* < 0.01. To calculate the average difference across all eight trials, we calculated the percent difference between conditions for each of the eight trials and then took the mean of these eight values.

To calculate differences in the rate of exploration by members of each team, we counted the number of choices to add or remove a variable by all members of a team across all rounds. This test was conducted at the group-level. We failed to reject the null hypothesis that there was no difference in the exploration rate with a probability of *P* = 0.95.

To examine the distribution of solutions across all members of a team, we constructed density plots in Figure 2 of the main text. While the time scales varied slightly in each competition, the general dynamics held across each competition (Figure S8). Initially, teams with efficient networks performed better than teams with inefficient networks because their best solution diffused rapidly to most of the team (Figure S8, A and B). By Rounds 5 and 10, efficient networks had not improved much from their initial solutions, but most inefficient networks had started to surpass the efficient networks (Figure S8, C and D). By the conclusion of every trial, teams with inefficient networks had found better solutions than teams with efficient networks (Figure S8, E).


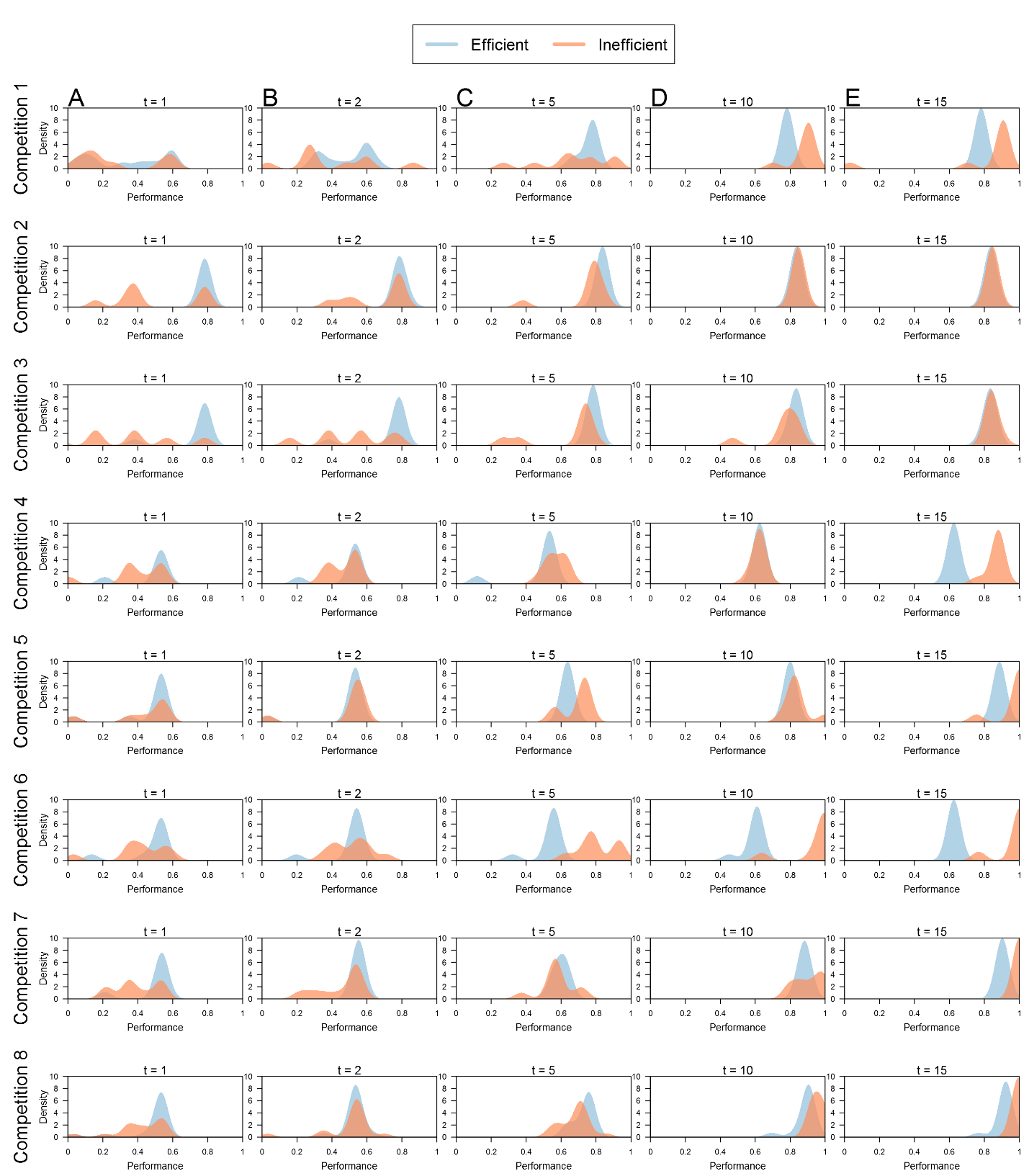


**S8 Fig. Solution quality at times *t* = 1, 2, 5, 10, and 15 (columns A to E) in all eight trials.**

To examine the rate of diffusion of top solutions in Figure 3A of the main text, we counted the fraction of individuals who adopted the best solution on every round following its discovery. This value was calculated for each team within each trial by finding the best solution for a team at time *t* and then counting the number of individuals who adopted that solution at time *t*+1. We then took the average count across all eight efficient networks and plotted that against the average count across all eight inefficient networks.

To examine the number of diverse solutions that were adopted in Figure 3B of the main text, we counted the cumulative number of unique solutions that were adopted by any member of a team up until time *t*. We then took the average number of unique solutions across all eight efficient networks and plotted that against the average number of unique solutions across all eight inefficient networks. To test the difference in the number of unique solutions that were found between teams with efficient and inefficient networks, we conducted a Wilcoxon signed-rank test. We rejected the null hypothesis that there was no difference in the number of unique solutions with a probability of *P* = 0.02.

To examine the performance of the best solutions in aggregate across all eight trials in Figure 3C of the main text, we found the best solution in each team at time *t* and then averaged this value across all teams with efficient networks and all teams with inefficient networks.

To examine how much better individuals performed in teams with inefficient networks compared to teams with efficient networks in Figure 3D of the main text, we calculated the number of individuals at time *t* in one team who had a solution that was better than the best available solution in the other team at time *t* for each trial. For the inefficient network, we counted the number of people at time *t* who had a better solution than the best solution available in the team with an efficient network at time *t*, and we did the same calculation for members of the team with the efficient network at time *t* compared to the best solution available in the team with an inefficient network at time *t*. We then averaged these values across all eight trials, and plotted it across time.

**Subject Retention.** To calculate attrition rates, we counted the number of participants who entered a response on the final round. This number is likely an over-estimation of attrition because if a subject thought that their current solution was the best option, they could choose not to submit a revision for that round, and have their previous solution counted. The experiment had a high retention rate, with 87% of all subjects entering a response on the final round. There was no significant difference in retention rates across conditions, with 86% of subjects finishing the study in inefficient networks, and 88% of subjects doing so in efficient networks (*P* = 0.75, Wilcoxon signed-rank test). The most common reasons for attrition were due to network connectivity issues, where the platform would disconnect a user if their browser stopped responding to our server, most likely because subjects closed their browser tab. We used the data from an individual until they left the study, or completed the final round.

**Ensuring Data Quality.** We took several precautions in order to ensure that subjects did not violate the design of the experiment. Such precautions can be more difficult in online experiments because researchers may have less control over the behavior of the subjects than in traditional laboratory settings. We took several steps to ensure that each participant was a unique user. In order to prevent individuals from participating in the study multiple times, we designed the system so that if a user tried to use a second browser tab to simultaneously participate, the system would produce an error, and only allow one active browser tab to communicate on the same computer. Additionally, we required users to enter their email address before playing the game, and all payments were sent to these addresses, which made it more difficult for users to gain access to the system multiple times. To do so, a user would have had to enroll with multiple email addresses. Even if users were able to bypass these measures, we only used a data problem for a short period of time so that repeat users would not have any advantage over new players. The interface was explained with a video instruction as users waited for the game to start, so there was very little reason to believe that there was any skill or learning that could occur from having played the game before.

**Supplementary Discussion**

**Incremental and Non-Incremental Search.** When individuals explored new solutions, they could make a single revision each round by adding one variable into or removing one variable from their model. This design permitted individuals to search locally by exploring solutions that were closely related to their present solution (i.e., incremental search). We chose this design because we wanted to capture realistic search processes by individuals and organizations in high-risk situations, where there are strong incentives to add slowly to a solution that has received heavy investment. Research into decision-making has shown that animals, individual humans, teams and organizations, and even many high performing artificial intelligence systems use greedy decision making that emphasizes incremental search as a method for global optimization (9–14).

To examine the sensitivity of the results to the design choices, we conducted agent-based simulations to examine how non-incremental, random leaps across the solution space might alter the results. We used populations of size 100, and allowed agents to search an NK space with N=14 and K=5, so that the problem spaces were similar to the ones used in our experimental study. We allowed the simulations to run for 50 rounds. The main parameter we explore is the frequency with which agents adopt a new solution by taking a non-incremental, random leap across the solution landscape (Figure S9). With some probability, agents could randomly choose another solution’s bitstring and adopt that solution regardless of whether it performed better than their current solution. Otherwise, agents would behave normally by copying any neighbor who was better than them, or exploring otherwise. This setup allowed populations to escape from local peaks to suddenly leaping to a new part of the solution space with some probability.

The simulation results show that the experimental results are not very sensitive to the design choice of encouraging incremental search (Figure S9). When agents make few random decisions, inefficient networks still outperform efficient networks. As agents make up to 50% of their decisions randomly, the difference between the networks becomes smaller because both populations are driven more by the dynamics of random search and less by any dynamics arising from the structure of the teams. The intuition that random noise in agents’ decisions will help efficient networks get off of local optima is partly true, as efficient networks perform best when agents make 5% of their plays as random leaps across the solution space. But additional noise beyond 5% only further degrades performance, as the size of the solution space makes random leaps less likely to be successful.


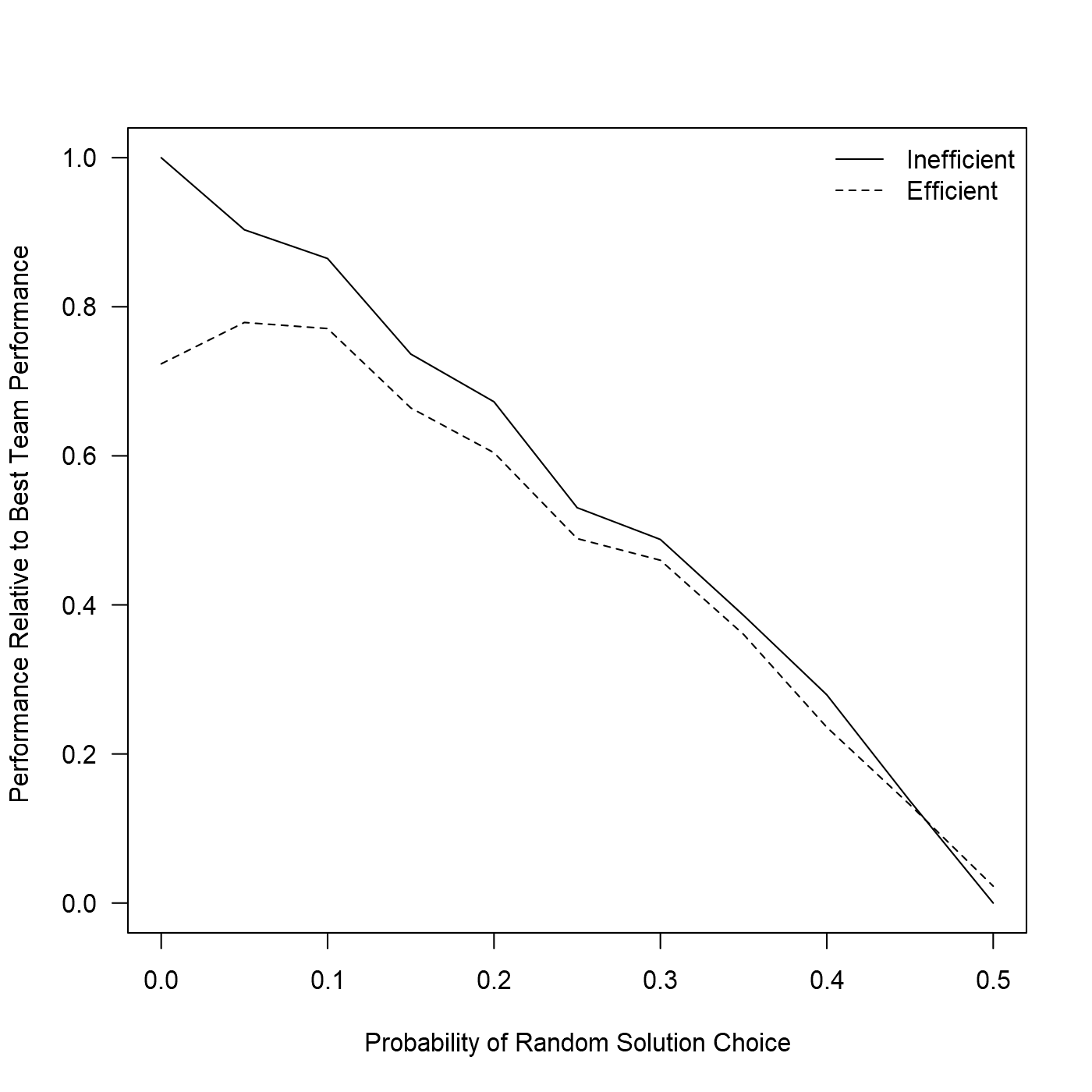


**S9 Fig. Simulations showing the effects of random noise in agents’ decisions on the performance of inefficient and efficient networks.** The performance of teams relative to the best group performance (i.e., 0 probability of making a random solution choice) is plotted against the probability of making a random choice on each turn.

To further examine the sensitivity of the results to this design choice, we altered the rules in Trial 7 so that individuals were permitted to make as many changes to their model on each round before submitting their new solution and receiving feedback (i.e., non-incremental search). The results in this trial were consistent with those in the other seven trials. At the individual level, individuals did attempt to explore more widely when given the option. 22% of all decisions were non-incremental searches. Based on the simulations in Figure S9, this is well within the parameter range of where the effect of network structure will still be influential on team performance. Across all attempts to explore in Trial 7, 38% of attempts were non-incremental searches. However, a majority of these attempts were unsuccessful, and individuals had a higher success rate when they explored incrementally. When incremental search was used, individuals successfully found a better solution 19.4% of the time, in contrast to a success rate of only 9.8% for attempts to change more than one component of their solution. This result is consistent with a level of random decision making that would show a difference between network structures in the simulations above, and it confirms the notion that individuals prefer incremental exploration in large part because exploring locally is more likely to provide a solution that is similar in quality to the current solution.

**Limitations.** Some of the design choices that aided the control of our *in vivo* study also put constraints on the behaviors that we could test. For instance, we were limited to a time length of 15 rounds. While longer experiments were possible, we found that efficient networks became locked into one of the local optima of the complex problem spaces within the length of our study. The only advantage to a longer duration study is that the inefficient networks that did not yet reach the optimum would be more likely to find it. Thus, our results offer a lower bound on the effectiveness of less efficient networks for team performance on relatively short timescales.

Notably, our study prevented participants from moving off from local peaks once they were found. An interesting direction for future research would be to examine how exogenous noise, or “simulated annealing”, could be used in a real world setting to help teams escape local optima and find better solutions (1).

**Supplementary References**

1. Lazer D, Friedman A. The Network Structure of Exploration and Exploitation. Adm Sci Q. 2007 Dec 1;52(4):667–94.

2. Shao J. An asymptotic theory for linear model selection. Stat Sin. 1997;7(2):221–242.

3. Kauffman S. The Origins of Order: Self-organization and Selection in Evolution. New York, NY: Oxford University Press; 1993. 740 p.

4. Friedman J, Hastie T, Tibshirani R. The elements of statistical learning. Vol. 1. Springer series in statistics Springer, Berlin; 2001.

5. Lichman M. UCI Machine Learning Repository [Internet]. Irvine, CA: University of California, School of Information and Computer Science; 2013. Available from: http://archive.ics.uci.edu/ml

6. Wasserman S, Faust K. Social Network Analysis: Methods and applications. Vol. 8. Cambridge University Press; 1994.

7. Imbens GW, Rubin DB. Causal inference for statistics, social, and biomedical sciences. Cambridge University Press; 2015.

8. Schwarz G. Estimating the dimension of a model. Ann Stat. 1978;6(2):461–464.

9. Cohen WM, Levinthal DA. Absorptive Capacity: A New Perspective on Learning and Innovation. Adm Sci Q. 1990 Mar 1;35(1):128–52.

10. March JG. Exploration and Exploitation in Organizational Learning. Organ Sci. 1991;2(1):71–87.

11. March JG, Simon H. Organizations. Cambridge, MA: Blackwell; 1958.

12. Nelson R, Winter S. An Evolutionary Theory of Economic Change. Cambridge, MA: Belknap Press; 1982.

13. Stuart TE, Podolny JM. Local search and the evolution of technological capabilities. Strateg Manag J. 1996 Jun 1;17(S1):21–38.

14. Eberhart RC, Shi Y, Kennedy J. Swarm Intelligence. Elsevier; 2001.
