# Supplementary figures and images for: Impact of network structure on collective learning: An experimental study in a data science competition
Source: PLoS One. 2020 Sep 4;15(9):e0237978. doi: 10.1371/journal.pone.0237978 (PMC7473554; doi:10.1371/journal.pone.0237978)

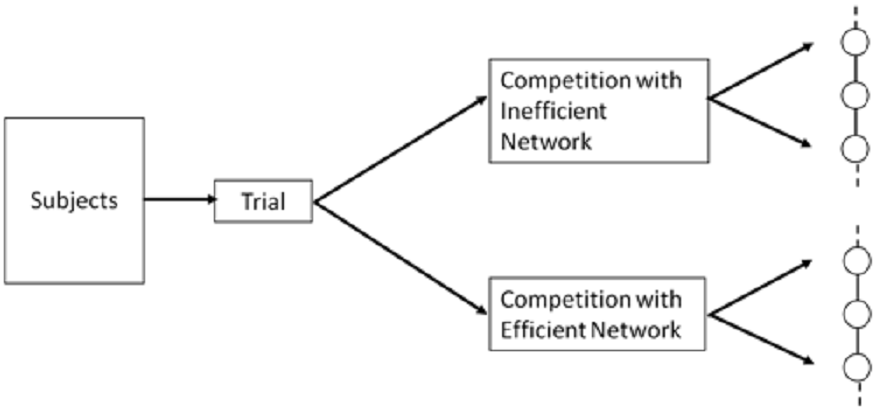

Supplement: S1 Fig — (TIF) [file pone.0237978.s002.tif]

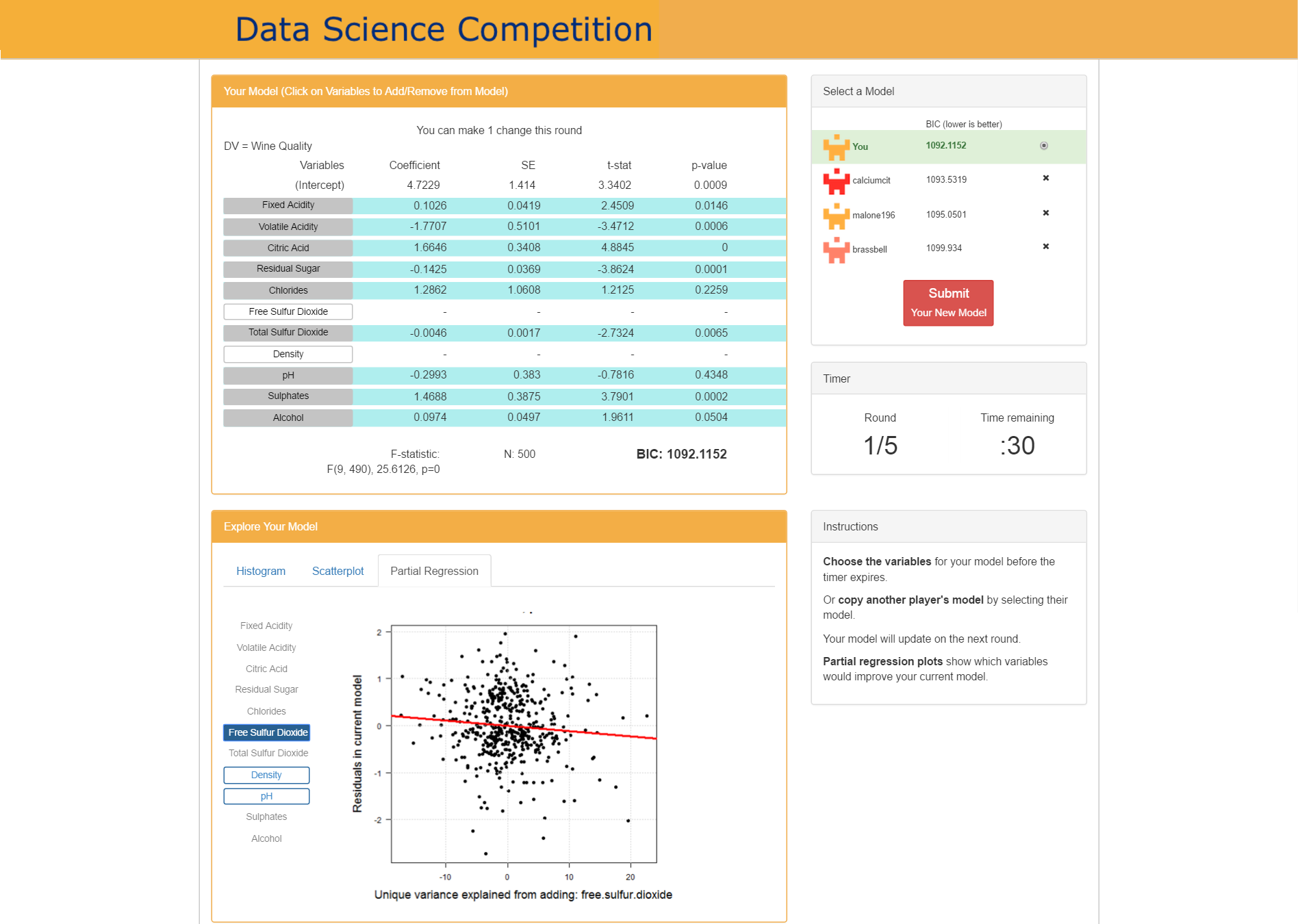

Supplement: S2 Fig — The image is similar but not identical to the experimental interface in that a university logo has been removed. (TIF) [file pone.0237978.s003.tif]

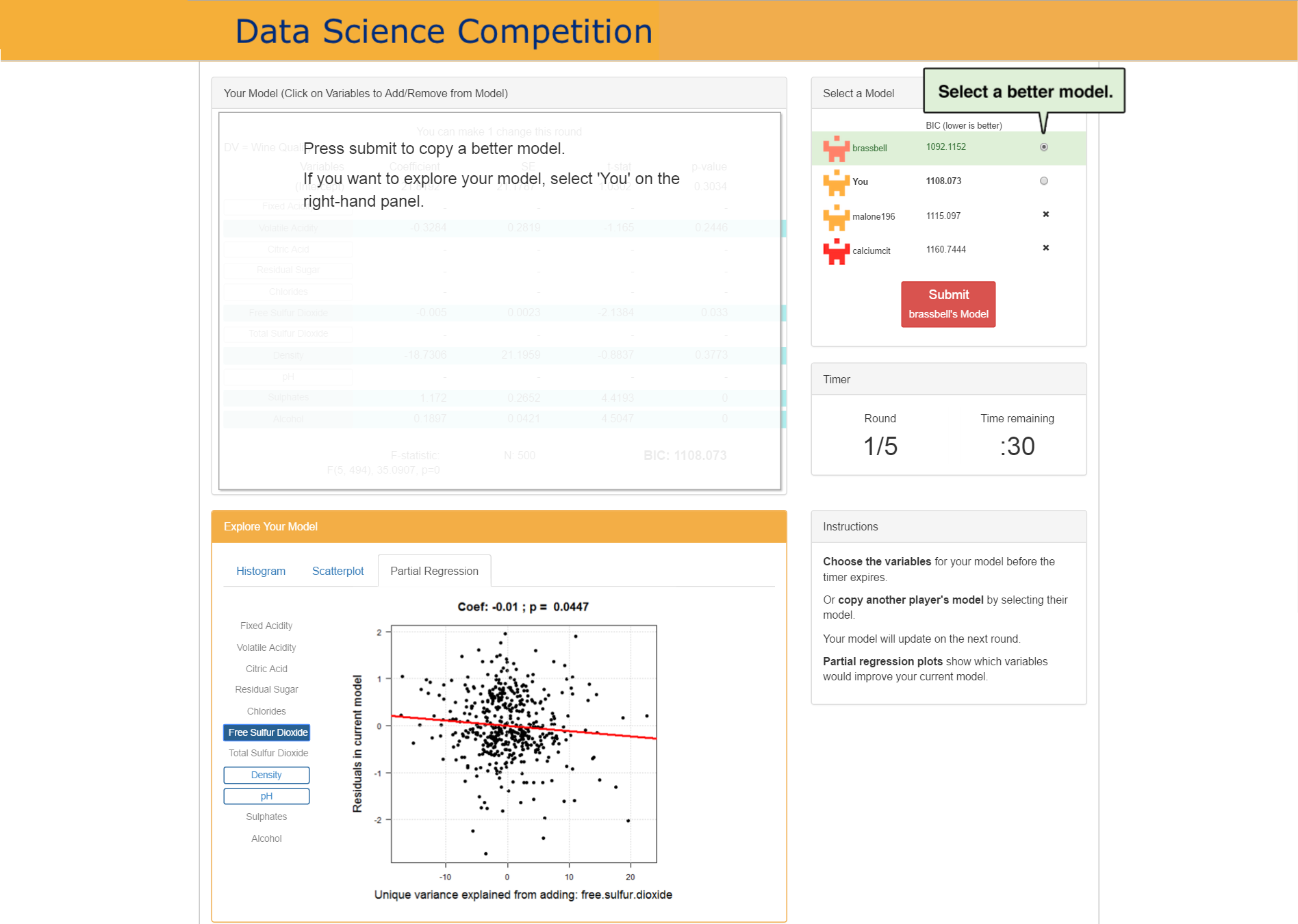

Supplement: S3 Fig — The image is similar but not identical to the experimental interface in that a university logo has been removed. (TIF) [file pone.0237978.s004.tif]

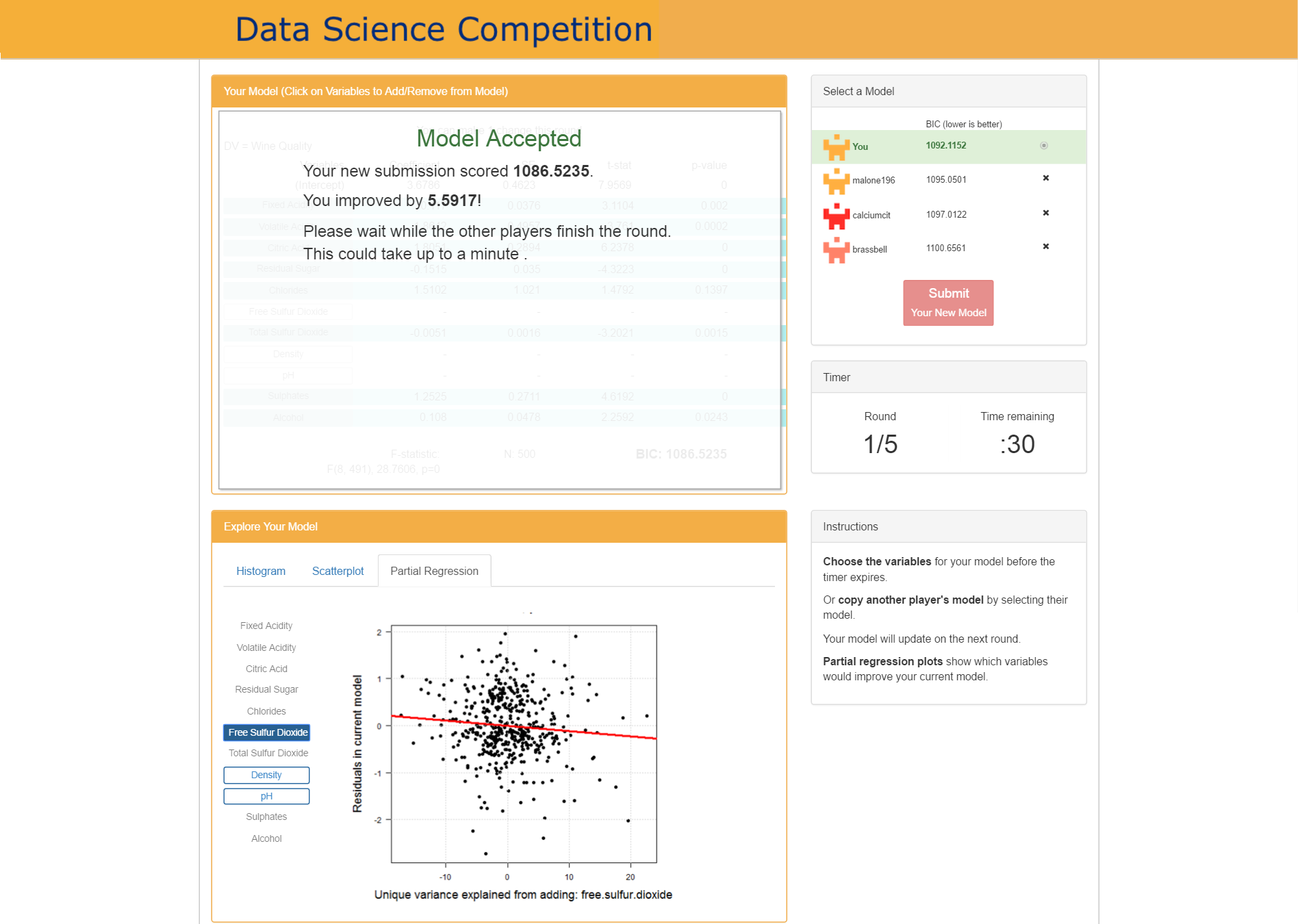

Supplement: S4 Fig — The image is similar but not identical to the experimental interface in that a university logo has been removed. (TIF) [file pone.0237978.s005.tif]

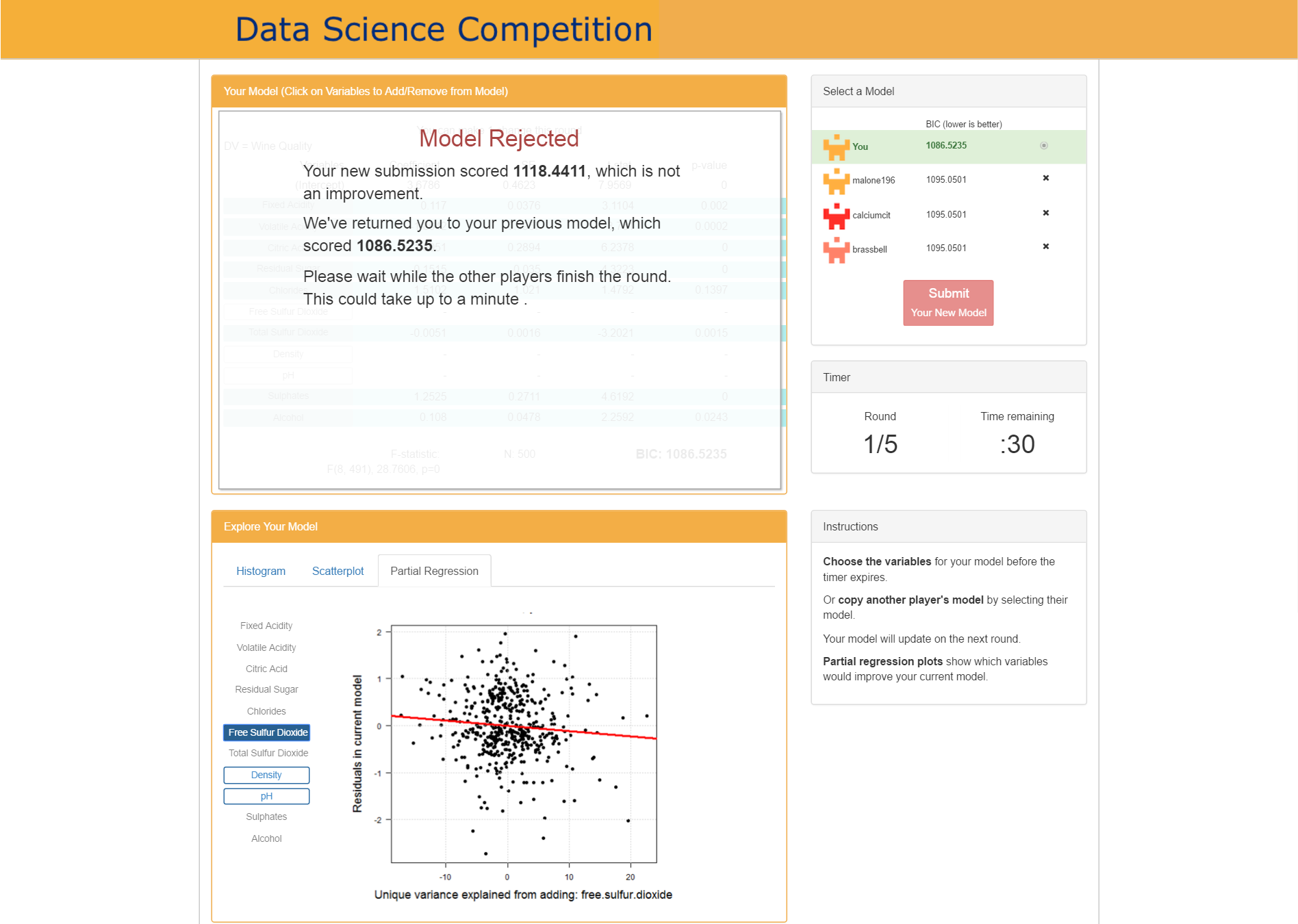

Supplement: S5 Fig — The image is similar but not identical to the experimental interface in that a university logo has been removed. (TIF) [file pone.0237978.s006.tif]

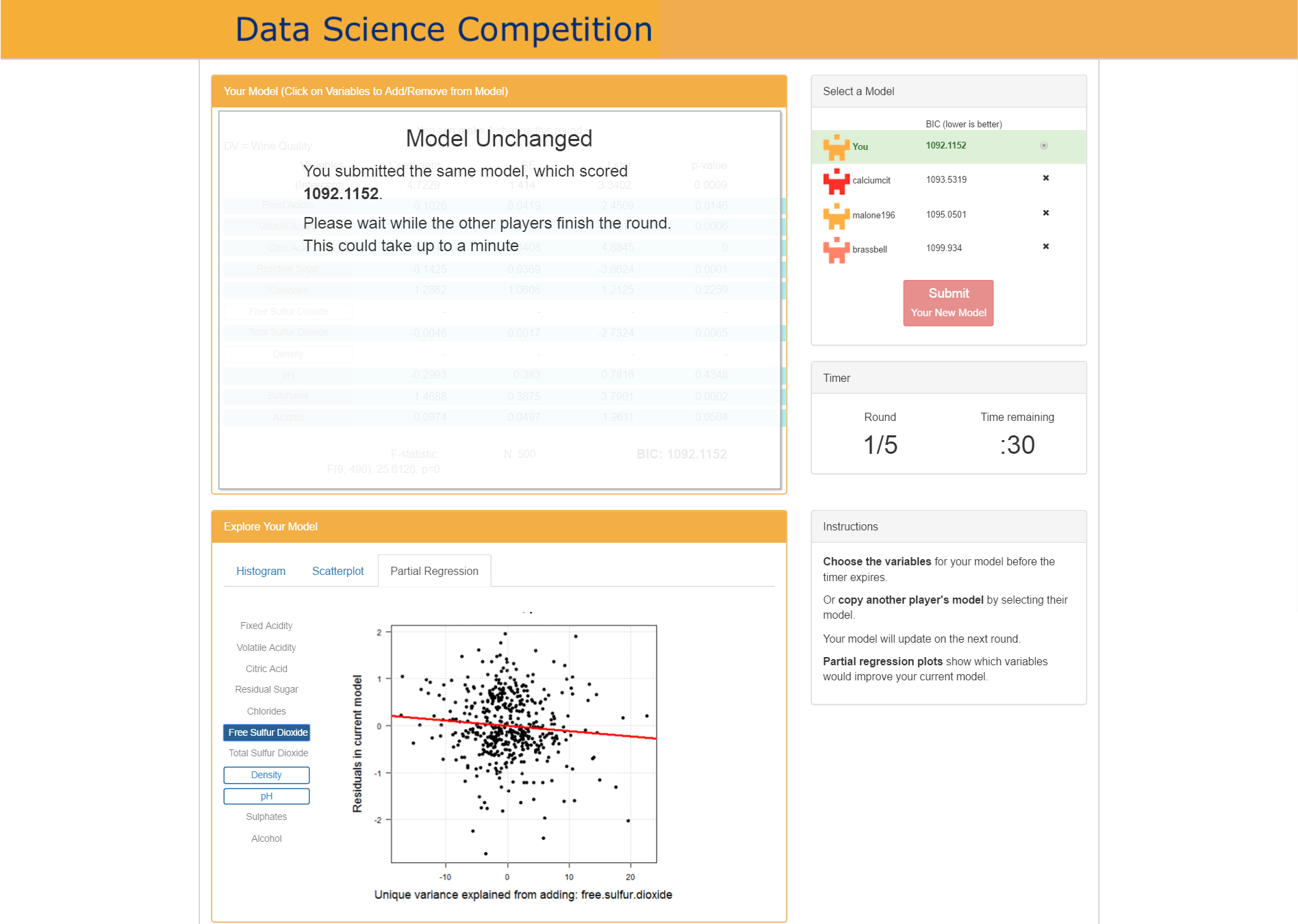

Supplement: S6 Fig — The image is similar but not identical to the experimental interface in that a university logo has been removed. (TIF) [file pone.0237978.s007.tif]

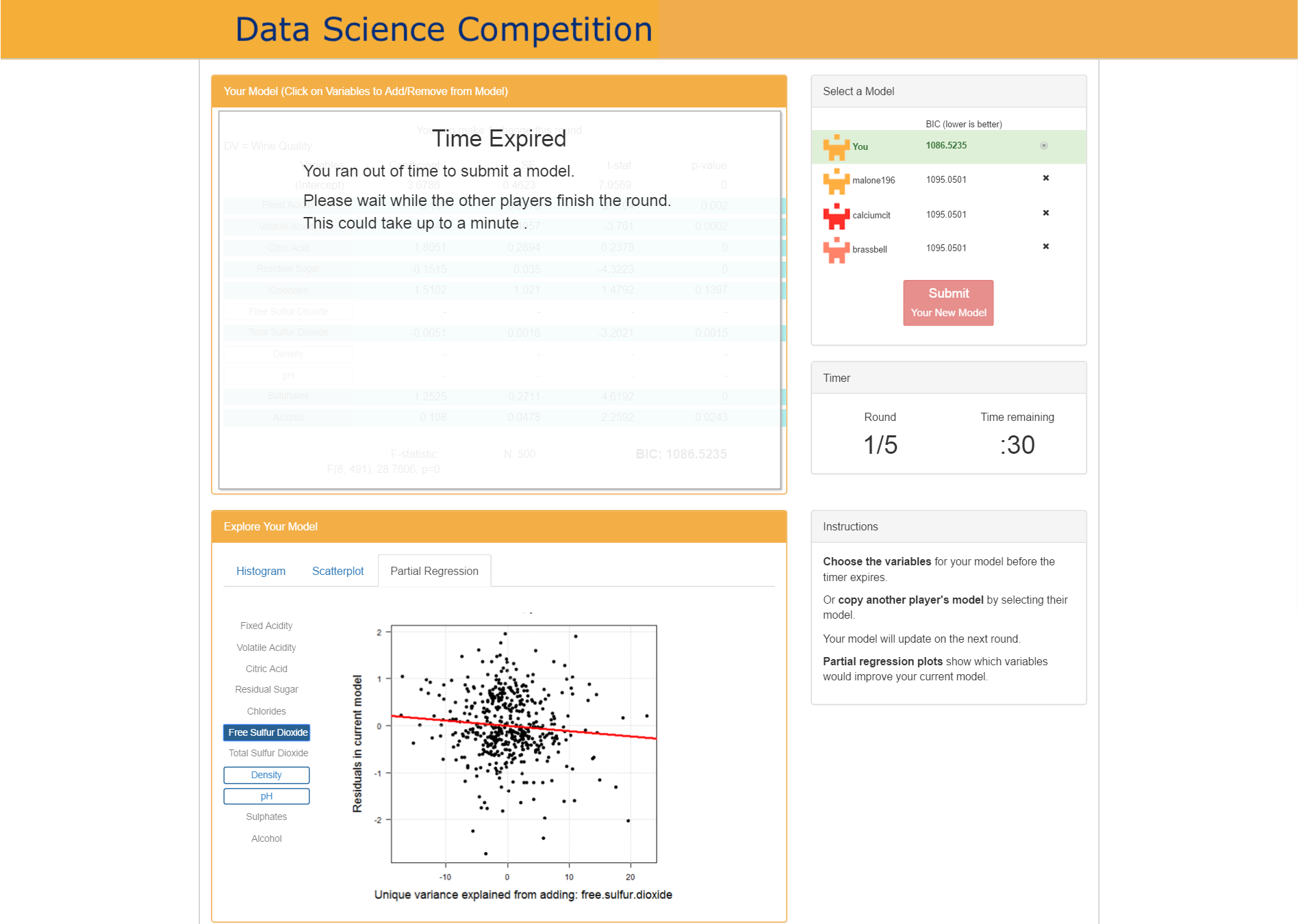

Supplement: S7 Fig — The image is similar but not identical to the experimental interface in that a university logo has been removed. (TIF) [file pone.0237978.s008.tif]

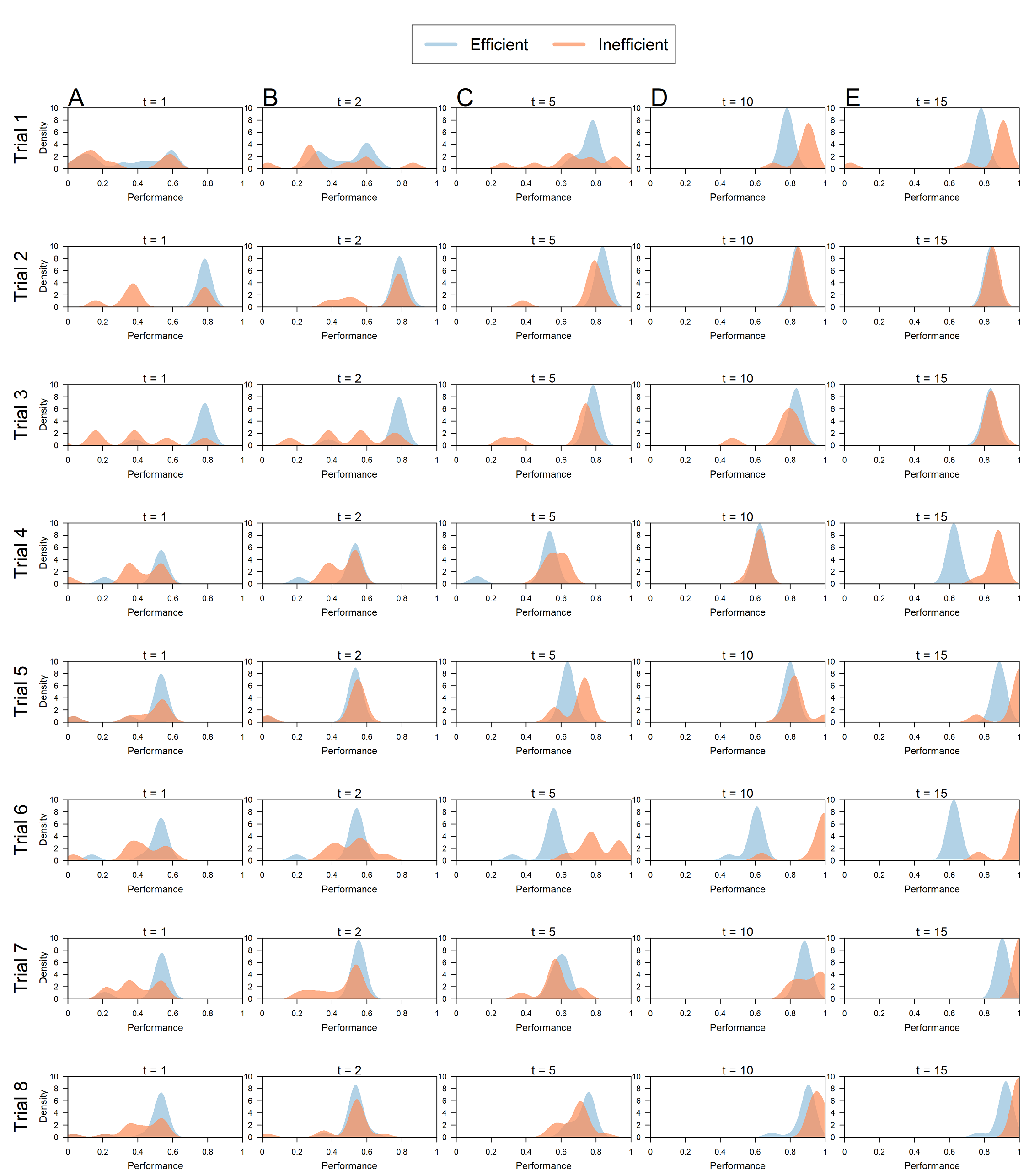

Supplement: S8 Fig — (TIF) [file pone.0237978.s009.tif]

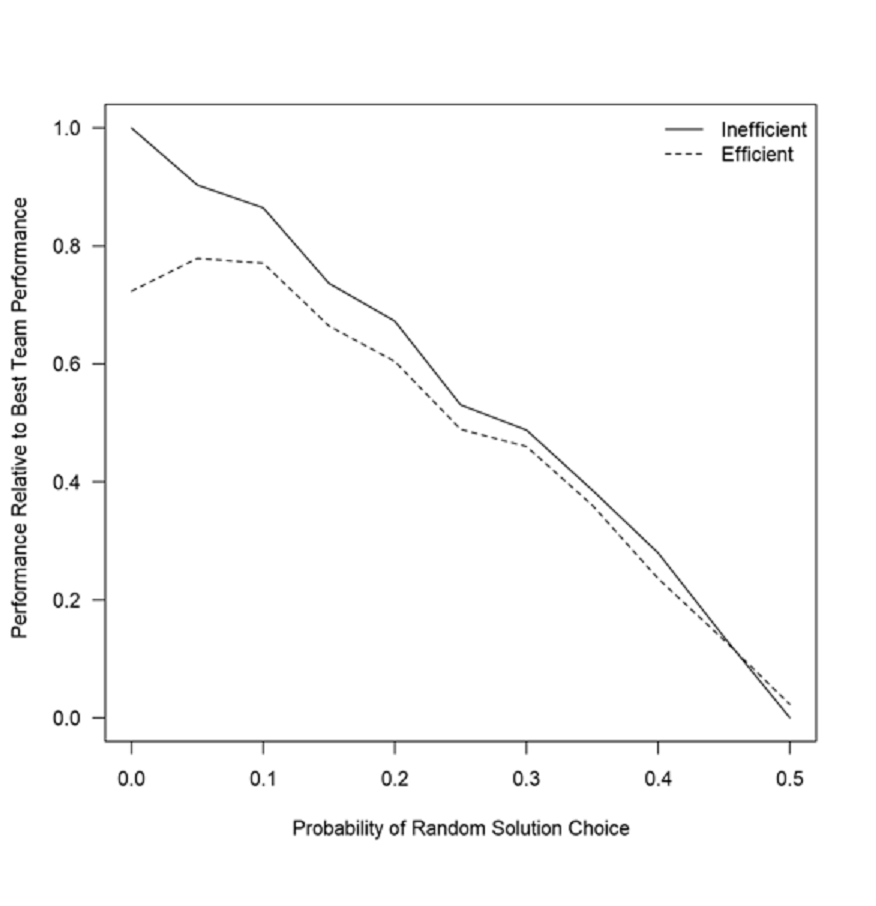

Supplement: S9 Fig — The performance of teams relative to the best group performance (i.e., 0 probability of making a random solution choice) is plotted against the probability of making a random choice on each turn. (TIF) [file pone.0237978.s010.tif]
