# Supplementary material for: Impact of network structure on collective learning: An experimental study in a data science competition
Source: PLoS One. 2020 Sep 4;15(9):e0237978. doi: 10.1371/journal.pone.0237978 (PMC7473554; doi:10.1371/journal.pone.0237978)
Supplement: S1 Table — (DOCX) [file pone.0237978.s011.docx]

|  | |  | | | | | |  |  |
| --- | --- | --- | --- | --- | --- | --- | --- | --- | --- |
| Competition | Data Set | | | Variables (no.) | Total Solutions (no.) | Variables in Optimal Solution (no.) | Local Optima  (no.) | Variables in Local Optima (mean) | Obs.  (no.) |
| 1 | Wine | | 11 | | 2,048 | 5 | 11 | 6.7 | 500 |
| 2 | Online News Popularity | | 14 | | 16,384 | 4 | 9 | 4.6 | 500 |
| 3 | Online News Popularity | | 14 | | 16,384 | 4 | 9 | 4.6 | 500 |
| 4 | Sales Forecast | | 14 | | 16,384 | 7 | 16 | 5.9 | 1000 |
| 5 | Sales Forecast | | 14 | | 16,384 | 7 | 16 | 5.9 | 1000 |
| 6 | Sales Forecast | | 14 | | 16,384 | 7 | 16 | 5.9 | 1000 |
| 7 | Sales Forecast | | 14 | | 16,384 | 7 | 16 | 5.9 | 1000 |
| 8 | Sales Forecast | | 14 | | 16,384 | 7 | 16 | 5.9 | 1000 |

S1 Table. Descriptive statistics of the data sets used in the study.
